# Revealing Intra- and Intermolecular Interactions Determining Physico-Chemical Features of Selected Quinolone Carboxylic Acid Derivatives

**DOI:** 10.3390/molecules27072299

**Published:** 2022-04-01

**Authors:** Kamil Wojtkowiak, Aneta Jezierska, Jarosław J. Panek

**Affiliations:** Faculty of Chemistry, University of Wrocław, ul. F. Joliot-Curie 14, 50-383 Wrocław, Poland; kamil.wojtkowiak@chem.uni.wroc.pl

**Keywords:** quinolone carboxylic acid, AIM, NBO, SAPT, CPMD, PIMD

## Abstract

The intra- and intermolecular interactions of selected quinolone carboxylic acid derivatives were studied in monomers, dimers and crystals. The investigated compounds are well-recognized as medicines or as bases for further studies in drug design. We employed density functional theory (DFT) in its classical formulation to develop gas-phase and solvent reaction field (PCM) models describing geometric, energetic and electronic structure parameters for monomers and dimers. The electronic structure was investigated based on the atoms in molecules (AIM) and natural bond orbital (NBO) theories. Special attention was devoted to the intramolecular hydrogen bonds (HB) present in the investigated compounds. The characterization of energy components was performed using symmetry-adapted perturbation theory (SAPT). Finally, the time-evolution methods of Car–Parrinello molecular dynamics (CPMD) and path integral molecular dynamics (PIMD) were employed to describe the hydrogen bond dynamics as well as the spectroscopic signatures. The vibrational features of the O-H stretching were studied using Fourier transformation of the autocorrelation function of atomic velocity. The inclusion of quantum nuclear effects provided an accurate depiction of the bridged proton delocalization. The CPMD and PIMD simulations were carried out in the gas and crystalline phases. It was found that the polar environment enhances the strength of the intramolecular hydrogen bonds. The SAPT analysis revealed that the dispersive forces are decisive factors in the intermolecular interactions. In the electronic ground state, the proton-transfer phenomena are not favourable. The CPMD results showed generally that the bridged proton is localized at the donor side, with possible proton-sharing events in the solid-phase simulation of stronger hydrogen bridges. However, the PIMD enabled the quantitative estimation of the quantum effects inclusion—the proton position was moved towards the bridge midpoint, but no qualitative changes were detected. It was found that the interatomic distance between the donor and acceptor atoms was shortened and that the bridged proton was strongly delocalized.

## 1. Introduction

**Figure 1:**
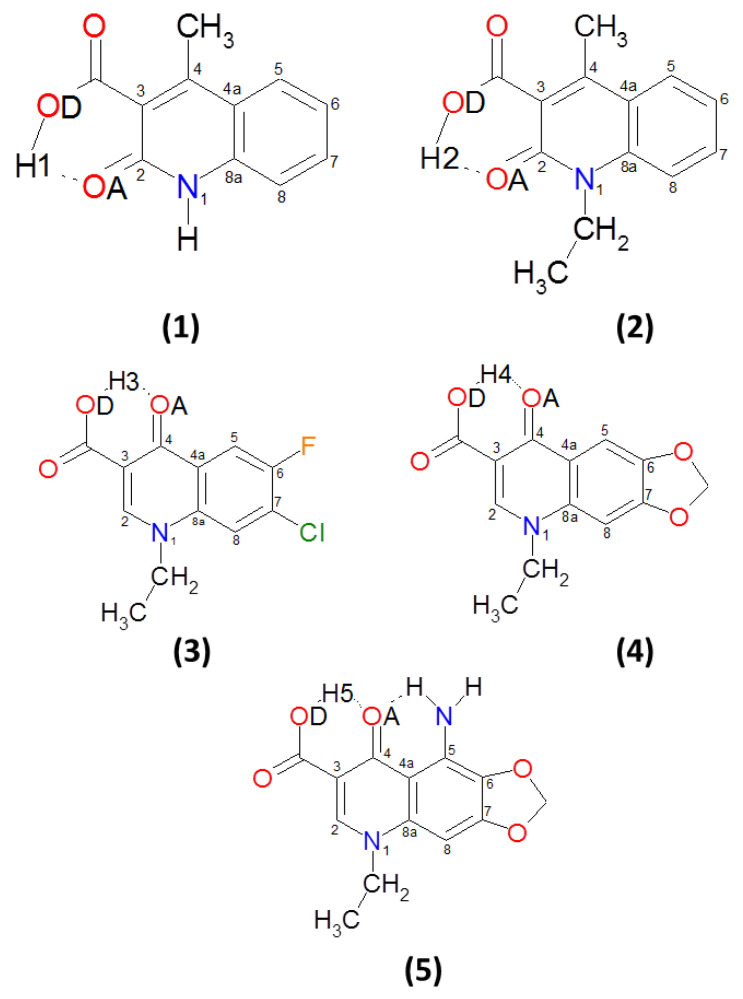
Molecular structures of the studied quinolone carboxylic acid derivatives. The dotted line indicates the presence of an intramolecular hydrogen bond. The OD indicates the proton-donor oxygen atom, while OA indicates the proton-acceptor oxygen atom. Hn indicates the bridged proton in the hydrogen bond.

Quinolone-based compounds are known for their biological activity, which makes them attractive for drug design and practical application in medicine [1,2,3,4,5,6,7,8]. A recent example is an effort in the area of “drug repositioning” that is finding new uses for the drugs currently used, abandoned or withdrawn from use: rare-earth complexes of oxolinic acid have shown cytotoxic activity towards diverse cancer cell lines [9]. A detailed description of physico-chemical features can significantly facilitate the rational design of new substances based on a quinolone structural motif. In the current study, five quinolone carboxylic acid derivatives were investigated on the basis of diverse quantum-chemistry approaches. The molecular structures of the discussed compounds are presented in Figure 1. They belong to 2- or 4-quinolone derivatives, where the carbonyl oxygen atom is involved in intramolecular hydrogen bond formation with the carboxylic OH group—one of the substituents. We chose quinolone carboxylic acid derivatives to be substituted by methyl, ethyl and amino groups, fluorine and chlorine atoms as well as 1,3-dioxolane. The latter substituent introduced an additional structural motif to the quinolone moiety resulting in oxolinic acid derivative formation (Compounds **4** and **5**, see Figure 1).

The presence of an intramolecular O–H•••O hydrogen bond resulted in quasi-ring formation and, as a consequence, structural stabilization. The investigated compounds could be considered as the basis for the synthesis of new potentially biologically active substances [10,11,12]. However, Compound **4**, oxolinic acid, has already found a practical application in medicine [13] and has been extensively studied [1,9,14,15,16]. Let us characterize the significant non-covalent interactions (NCIs), in particular intra- and intermolecular hydrogen/halogen bonds, present in the investigated compounds. Compound **1** [10] can form, beside intramolecular O–H•••O hydrogen bond, an N–H•••O intermolecular hydrogen bond. The oxygen atom from the carbonyl group of the carboxylic moiety was found to be the proton acceptor. In Compound **2** [17], an intermolecular C–H•••O hydrogen bond was found as well. Similarly to Compound **1**, the carbonyl group oxygen atom from the carboxylic moiety is involved in its formation. In the case of Compound **3** [18], C–H•••O and C–H•••Cl bonds were detected in the crystal structure. They are involved in the molecular connectivity pattern of two-dimensional layers. In Compound **4**, a bifurcated hydrogen bond is present [11]. According to the experimental data, a weak intermolecular hydrogen bond was formed with an adjacent molecule from the same proton donor. Compound **5** possesses two intramolecular hydrogen bonds, O–H•••O and N–H•••O, where the carbonyl oxygen atom from the quinolone moiety serves as a proton acceptor in both cases [12].

Non-covalent interactions (NCIs) play a crucial role in the binding between receptors and ligands, in the stabilization of the tertiary structure of proteins, in DNA helices and also in crystal engineering or enzymatic catalysis processes [19,20,21,22]. Moreover, hydrogen bonds and other interactions between Lewis acids and Lewis bases are also recognized as a preliminary stage of chemical reactions with the S_N_2 mechanism. In the case of NCIs, significant electron charge shifts occur (and the covalency of the bond is strongly associated with these shifts); other names, such as secondary bonding [23], have therefore been suggested and can be regarded as more suitable. One of the most important examples of NCIs is the hydrogen bond (HB). It is considered as electrostatic (and also, to some extent, covalent, especially in cases of strong HBs) in nature and can be defined as an interaction of an electronegative atom (proton donor) through the hydrogen that is attached to it with another, relatively electronegative atom (proton acceptor) [24]. Numerous studies have shown that the strength of the HB can vary significantly and that it is closely associated with proton-transfer processes, e.g., [19,25,26,27,28,29,30,31]. The so-called weak hydrogen bonds are usually composed of carbon atoms as proton donors C–H•••X or proton acceptors X–H•••C, whereas strong hydrogen bonds are often classified into charge-assisted hydrogen bonds (CAHB) or resonance-assisted hydrogen bonds (RAHB) [32]. Another classification can be made on the basis of Pauling criteria for the electronegativity of atoms that are involved in the hydrogen bond formation as typical and non-conventional H-bonds, wherein both the former proton acceptor and the former proton donor satisfy Pauling’s criteria, whereas for the latter carbon as proton donor and an aromatic ring as proton acceptor can be present [33]. The other similar kind of NCI is the halogen bond, a type of sigma-hole interaction, which arises due to the anisotropic distribution of electron density around the covalently bonded halogen atom [34,35]. In the case of halogens, the sigma-hole usually exists on the extension of the covalent bond and is associated with a positive (or less negative) electrostatic potential. Interestingly, halogens also possess an increased electron charge density in the direction perpendicular to the bond axis, which accounts for their Lewis base properties and forms the so-called belt of negative electrostatic potential around it. Generally, these types of interaction can be described as R–X•••: A, where X is any element from the 17th group in the periodic table [36]. In terms of interaction energy, they can be classified as only a little less strong than HBs, and their strength grows as polarizability of the halogen atom increases. Among further important factors that have an impact on halogen bond energy is the presence of electron-withdrawing groups near the halogen atom—the larger the electron-withdrawing effect, the more positive the electrostatic potential hole and the stronger the interaction of the halogen with the electron-rich entity [37,38,39,40]. Splendid examples of these effects are illustrated in the case of CH_3_Cl and CF_3_Cl molecules, where, in the absence of fluorine atoms, the σ-hole on the chlorine atom does not even exist [41,42].

Therefore, the main aims of the current study are related to the investigation and understanding of the non-covalent interactions present in the selected quinolone derivatives. Beside intramolecular hydrogen bonds present in these compounds, C–Cl•••H–C, C–Cl•••O and C–F•••F NCIs have been discussed as well. Finally, weaker interactions, e.g., dispersion and electrostatics, were also taken into account. In order to prepare an accurate description of the interactions and draw conclusions on the physico-chemical features of the investigated quinolone carboxylic acid derivatives, quantum-chemical simulations were carried out for monomers, dimers and crystals. We developed gas-phase, solvent reaction field as well as solid-state models to build up a database of selected interactions associated with the state of matter.

We have applied density functional theory (DFT) [43,44] to describe the molecular structures of monomers and dimers. The electronic structure and topological analyses were performed with assistance of the atoms in molecules (AIM) [45] and natural bond orbitals (NBO) [46,47,48] theories. The energy decomposition of dimers was prepared within symmetry-adapted perturbation theory (SAPT) [49]. Finally, time-evolution methods, Car–Parrinello molecular dynamics (CPMD) [50] and path integral molecular dynamics (PIMD) [51,52] were employed to shed light on the dynamical nature of the bridged protons and the spectroscopic properties associated with the analysed quinolone derivatives. To the best of our knowledge, this is the first comprehensive, quantum-chemical study discussing non-covalent interactions based on diverse theoretical approaches and in the three states of matter.

## 2. Computational Methodology

### 2.1. Density Functional Theory (DFT) Procedure for Monomers and Dimers

The models of the monomeric forms of the investigated quinolone carboxylic acid derivatives were prepared on the basis of their crystal structures [10,11,12,17,18] deposited in the Crystallographic Cambridge Data Centre (CCDC) [53]. The molecular forms of the investigated compounds are presented in Figure 1. The Mercury program [54] was used for structure visualization and geometries preparation for further quantum-chemical simulations. The closed (with intramolecular hydrogen bonds) and open forms of the monomers of the studied compounds were analysed. The simulations were performed in vacuo and using the continuum solvation model (IEF-PCM formula) [55,56], with water as a solvent. Density functional theory (DFT) [43,44] was applied for energy minimization. The Perdew–Burke–Ernzerhof [57] functional with dispersion correction according to Grimme [58] denoted as PBE-D3 was applied. Finally, the Dunning-type triple-zeta split-valence aug-cc-pVTZ basis set [59] was used to represent the electronic wave function of the investigated derivatives. Subsequently, the harmonic frequencies were computed to confirm that the obtained structures corresponded with the energy minima on the potential energy surface (PES). No imaginary frequency was detected. In the next step, the wavefunctions for atoms in molecules (AIM) [45] analysis were prepared. This part of the simulations was carried out using the Gaussian 16 program [60]. The AIM formalism was applied for the optimized structures with equilibrium and non-equilibrium geometries (in the case of dimers). The electronic structure (partial atomic charges and electron density and its Laplacian at bond and ring critical points (BCPs and RCPs, respectively)) and the topology of the quinolone derivatives were analysed based on the AIM theory. This part of the simulation was carried out using the AIMAll program [61]. Finally, natural bond orbital (NBO) [46] computations were performed. On the basis of the method, the partial atomic charges, interaction energy and population analysis including Wiberg bond indices [62] for the set of the studied compounds were obtained. The NBO 3.1 [63] and NBO 6.0 [64] programs incorporated in the Gaussian 09 Rev. C.0.1 [65] and Gaussian 16 Rev. A.0.3 [60] suite of programs were employed. The dimers of the quinolone derivatives (presented in Section 3.6) were prepared using the available X-ray data as mentioned above in the case of the monomers. The PBE-D3/aug-cc-pVDZ [57,58,59] level of theory was applied for the structure minimization and harmonic frequency computations. The simulations were performed in the gas phase. As a result, a set of geometries corresponding with the minima on the PES was obtained. The Gaussian 16 Rev. A.0.3. suite of programs [60] was used in this part of the computations. The graphical presentation of the NBO results was prepared with the assistance of the 14.6.4 Jmol software [66] in conjunction with the 2.1 version of the Jmol NBO Visualization Helper developed by Marcel Patek [67]. In the next step, the dimers’ interaction energy was estimated based on the symmetry-adapted perturbation theory (SAPT) [49].

### 2.2. Symmetry-Adapted Perturbation Theory (SAPT) Procedure for Dimers

Symmetry-adapted perturbation theory (SAPT) [49] was employed for the energy decomposition in the studied dimers of quinolone carboxylic acid derivatives (depicted in Section 3.6). Two sets of structures were taken into consideration:

(I) Extracted from the available X-ray data [10,11,12,17,18];

(II) Optimized at the PBE-D3/aug-cc-pVDZ level of theory in the gas phase.

The energy partitioning was performed at the SAPT0/aug-cc-pVDZ and SAPT2/aug-cc-pVDZ levels of theory [68,69]. In order to approximate the four-index integrals, the density fitting (resolution of identity (RI) and Coulomb and exchange integrals (JKI)) method with the same basis sets as mentioned above was employed. The Basis set superposition error (BSSE) was taken into account by separating the dimer into two monomers to fulfil the conditions needed to apply the Boys–Bernardi method [70]. The calculations of the SAPT interaction energies were performed using the Psi4 1.3.2 software [71].

### 2.3. Car–Parrinello Molecular Dynamics (CPMD) in Vacuo and the Crystalline Phase

The dynamical aspects of the molecular properties of the studied quinolone carboxylic acid derivatives were studied using Car–Parrinello molecular dynamics (CPMD) [50]. Initial structures for the gas-phase and solid-state calculations were taken from the experimental crystal structures [10,11,12,17,18]. An initialization of the Hessian matrix was employed in accordance with the Schlegel scheme [72] and the quasi-Newton method with a convergence criterion set to 5 × 10^−4^ was used to perform the structure optimization run. The gas-phase simulations were performed for individual molecules placed in cubic boxes of a = 20 Å edge, while the experimental crystal unit cells, summarized in Table 1, were used in the solid-state studies. For the gas-phase simulations, the Hockney method [73] was adopted to prevent interactions between neighbouring images of the molecule, whereas, for the solid state, the periodic boundary conditions (PBCs) with the eight nearest neighbours (TESR = 8) in every direction were applied. The CPMD was performed using the PBE exchange-correlation generalized gradient approximation (GGA) functional [57] in conjunction with the D2 Grimme empirical corrections for dispersion [58]. A canonical ensemble with electron fictitious mass parameter, time step and temperature equal to 400 a.u., 3 a.u. and 297 K, respectively, was applied during the simulation. The Nosé–Hoover chains algorithm [74] was employed to rescale velocities and maintain the assigned conditions. The plane wave basis set was truncated at a 100 Ry kinetic energy cut-off and the Troullier–Martins [75] norm-conserving atomic pseudo-potentials were chosen to represent the core electrons. The trajectories of 87 ps were obtained and analysed with scrutiny with the 1.9.3 and 5.2 versions of the VMD [76] and the gnuplot [77] set of programs, respectively. The initial parts of the CPMD trajectories were treated as an equilibration phase (10,000 steps) and thus were excluded from further analyses. This part of the study was carried out with the 4.3 version of the CPMD [78] code. The post-processing of the CPMD trajectories was devoted to the studies of metric parameters and spectroscopic features. Fourier transformation of the autocorrelation function of atomic velocity was applied for the analysis of spectroscopic signatures. The decomposition of the power spectra of atomic velocity was conducted using home-made scripts written in the Fortran programming language [79].

### 2.4. Path Integral Molecular Dynamics (PIMD) in Vacuo and the Crystalline Phase

Path integral molecular dynamics (PIMD) [51,52] was employed to investigate the quantum effects on the nuclear motions. The simulations were performed for the isolated molecule and in the crystalline phase. The electronic structure setup as described above concerning the CPMD runs (see Section 2.3) was applied, including Grimme D2 dispersion corrections to the PBE exchange-correlation functional and the plane-wave cut-off of 100 Ry. For the imaginary time path integration, 8 Trotter replicas (P = 8) were used. The PIMD trajectories were collected for 20 ps after the initial equilibration of 10,000 steps. This part of the simulations was carried out with the CPMD program, version 4.3 [78]. The post-processing of the PIMD trajectories was performed using home-made scripts and the gnuplot program [77].

## 3. Results and Discussion

### 3.1. Structural, Energetic and Electronic Aspects of Intramolecular Hydrogen Bonds in the Investigated Quinolone Carboxylic Acid Derivatives

The calculated metric parameters of the intramolecular hydrogen bonds are presented in Table S1. They are compared with the available experimental X-ray data [10,11,12,17,18]. The molecular structures of the studied compounds are presented in Figure 1 and Figure S1. As shown, the OD•••OA interatomic distance was the longest in the gas phase compared to the X-ray and computed data obtained from the application of the continuum solvation model PCM. The OD–Hn (where n is the number denoting the examined proton) covalent bond length was reproduced correctly by the theoretical approaches, and it was equal to ca. 1 Å. The X-ray data [10,11,12,17,18] reported the OD–Hn covalent bond length as being between 0.8 Å–0.95 Å depending on the compound. Comparing the DFT results obtained in the gas phase with those obtained in the solvent, it is clear that the discussed metric parameters obtained in the gas phase were slightly shorter. The results collected in Table S1 for the intramolecular hydrogen bonds show that the presence of the polar environment shortened them compared to the gas-phase results. However, the comparison of the gas-phase results with the X-ray data show that the hydrogen bond length obtained based on DFT was shorter. The bond angle values formed by the atoms involved in intramolecular hydrogen bond formation are listed in Table S1. The comparison of the theoretical results obtained in the gas phase with those obtained in the presence of the polar solvent show that the values were larger with the polar environment. Another case is the N–H•••OA intramolecular hydrogen bond present in Compound **5**. The experimental and computed data for the hydrogen bond are presented in Table S1 as well. The theoretically obtained interatomic distance N•••OA corresponds well with the X-ray result [12]. The difference between the experimental and the computed gas-phase data was equal 0.03 Å. The N–H covalent bond was reproduced correctly by the theoretical approaches, and it was equal to ca. 1 Å. The experimental H•••OA hydrogen bond was equal 1.978 Å [12], while the theoretically obtained bond lengths were shorter, and they were equal 1.8343 Å and 1.8819 Å for the gas-phase and PCM results, respectively. The bond angle values obtained based on the theoretical approaches was smaller compared to those obtained from the X-ray data. The differences between the experimental and the theoretical values were ca. 3.5${}^{\circ }$ and 4.8${}^{\circ }$. In conclusion, it was shown that the applied PBE-D3/aug-cc-pVTZ level of theory correctly reproduced the metric parameters of the intramolecular hydrogen bridges.

### 3.2. Intramolecular Hydrogen Bonds—Energy Estimation

The ultimate aim of the characterization of hydrogen bonds through structural or spectroscopic features is the assessment of their strength. In the case of intramolecular contacts (the issue is not limited to HBs), one encounters the problem of the proper definition of the systems between which the energy difference is calculated [30]. For the studied quinolone carboxylic acid derivatives **1**–**5**, the simplest approach, that is, the use of “open” and “closed” conformers differing by rotation of the HB-donating –OH group, seems to introduce minimal errors in the gas phase. The reason for this is that the HB proton in the gas-phase “open” conformation does not form any contacts potentially influencing the estimated HB energy. The results of the HB energy estimation via the open–closed approach are presented in Table 2.

The main factor determining the intramolecular HB strength is the relative position of the carbonyl group and the nitrogen heteroatom. The ortho arrangement in **1**–**2** led to weaker hydrogen bonds than the para arrangement of **3**–**5**. The comparison of the HB energies of **1** and **2** allows for the estimation of the steric strengthening of the HB. While there are systems in which steric repulsion significantly strengthens the hydrogen bonding [80], in the case of the studied quinolone derivatives there was virtually no impact of the presence of the ethyl group at the heterocyclic nitrogen atom of **2**—even worse, the HB is not as strong as in **1**. Furthermore, the impact of the heterocyclic oxygen-bearing ring in **4** and **5** was stronger than the substituent effect of –F and –Cl in **3**. Finally, the presence of an amino group in **5**, which forms another hydrogen bond with the OA carbonyl oxygen atom, weakened the OD–H5•••OA interaction. This weakening might have resulted for two reasons: an actual weakening of the H5•••OA bond, or a lowering of the energy of the open conformer due to the strengthening of the N–H•••OA bridge when the OD–H5 competitor was not present. The impact of the PCM solvation on the intramolecular HB energies was not large, but it was unmistakable: the HB was stronger in the polar medium. The most prominent increase was registered for Compound **5**. It is worth noting that the HB energy estimation in Table 2 is in good agreement with the available ^1^H NMR data. The chemical shifts for the bridge proton followed the changes in the HB energies: 13.84 ppm for **1** [81], 13.39 ppm for **2** [82] and 14.4 ppm for **3** [83].

### 3.3. Atoms in Molecules (AIM) Analysis of the Electronic Structure

**Figure 2:**
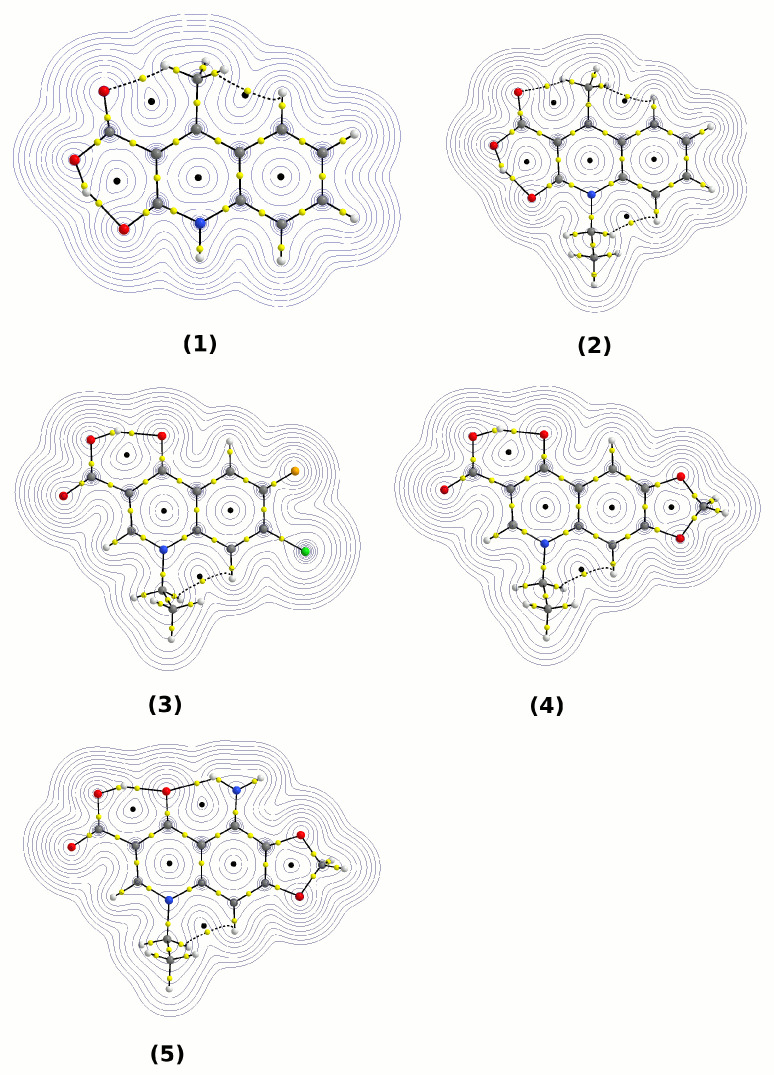
Topological maps of electron density based on AIM theory. Small yellow spheres denote bond critical points (BCPs), small black spheres denote ring critical points (RCPs). Atom colour coding: oxygen atoms, red; hydrogen atoms, white; carbon atoms, grey; chlorine atom, green; fluorine atom, orange; and nitrogen atoms, blue.

The atoms in molecules (AIM) theory [45] has been used to investigate the details of intramolecular hydrogen bonds formed within a quinolone-based molecule between the carboxylic and carbonyl groups. Applications of AIM to hydrogen bonding range from determining its existence [84], to investigating its nature [24] or assaying its strength [85]. From the topological point of view, an investigation within the framework of the AIM theory begins with the location of the relevant bond, ring and cage critical points (denoted as BCP, RCP and CCP, respectively). All the five studied quinolone derivatives possess relatively strong intramolecular hydrogen bonds between the carboxylic and carbonyl groups, and the topological search of the electron density field (see Figure 2) also reveals an N–H•••O bond for Compound **5** and weaker interactions of the C–H•••O type for Compounds **1** and **2**. These bonds are stronger than the very weak C–H•••H contacts present in all the structures—the C–H•••H contacts are characterized by the BCP and RCP located in very close proximity, close to coalescence, which is regarded as a sign of the structural instability of such interactions [84]. On the other hand, the OD–H•••OA bonds (and the N–H•••OA contact in **5**) exhibit very distinct separation between the corresponding BCP and RCP locations.

The parameters of the hydrogen bridge BCP, as well as the donor–proton covalent BCP, are related to the strength of the hydrogen bonding. There is a dispute concerning the use of AIM-derived energetic parameters for intramolecular hydrogen bonds [30]; therefore, we will base our discussion on the electronic structure descriptors—in particular, the electron density and its Laplacian at the relevant BCPs. The data in Table S3 indicate that the OD–Hn•••OA hydrogen bridges in Compounds **1**–**5** are of middle strength, in agreement with the estimations from Table 2. A comparison of the electron densities $\rho_{BCP}$ at the BCPs of the donor–proton and proton–acceptor bonds shows that the non-covalent hydrogen bond BCP has an electron density only 4–5 times lower than the covalent OD–H BCP. This apparent strengthening of the hydrogen bond is partially caused by the participation of this bond in the six-membered quasi-ring. Two edges of this quasi-ring are formed by aromatic (phenyl) or double (carbonyl) bonds, and when a typical bond alteration scheme is involved, the intramolecular hydrogen bond should also have some “double” (and, in reality, simply stronger) character. The Laplacian values at the H•••OA BCP, however, corresponded to the non-covalent, closed-shell type [86]. The most intriguing feature of the data in Table S3 is the sensitivity of the electronic structure parameters with respect to the presence of solvent. The inclusion of solvent effects, such as the mutual polarization of the solute and solvent, via the PCM model resulted in significant weakening of the donor–proton OD–H bond with simultaneous strengthening of the hydrogen bond. The impact of solvent was more pronounced for the weaker interaction, the hydrogen bond. While the electron density at the OD–H BCP decreased by ca. 6% when the PCM water model was employed, the H•••OA BCP increased its $\rho_{BCP}$ by 20–30%. The changes in the electron density were also reflected in its Laplacian. While the covalent OD–H bond was weakened on solvation (the Laplacian became less negative), the HB increased its covalency (the corresponding Laplacian became less positive, moving towards the negative values associated with covalency). This result indicates that molecular structures with separated charges, such as zwitterions or proton-transferred forms, are preferred in the polar environment because of the increased role of the stabilizing solute–solvent mutual polarization. The notion of the increased covalent character of the hydrogen bond upon solvation is also clear in the study of Wiberg bond indices in the following sections.

### 3.4. Natural Population Analysis (NPA)

Calculating the partial atomic charges can provide valuable insight into the studied structures, because it can provide information about the electron distribution throughout the molecule and help researchers to access molecular reactivity. Natural population analysis (NPA) is a wavefunction-based method that is considered to be less dependent on the basis set than the method of Mulliken [87]. Moreover, as studies have shown, with increasing basis set, the convergence of the atomic charge values for NPA can be observed [88]. Another method for calculating partial charges is the AIM theory, which is based on the physically observable, namely, the electron density. In order to shed light onto the charge distribution within the examined molecules, these two different approaches in the gas phase and in the polarizable continuum model (PCM) were employed (see Table 3 for the NPA; AIM was employed only for closed conformation, and the results are shown in Table S2 of the Supplementary Material).

The data gathered in Table 3 suggest strongly that the OA oxygen atom, which is an acceptor of the hydrogen bond, possesses lower atomic charge in the closed conformation than in the open one (which can be observed for the systems in the gas phase and PCM). Taking into consideration that the OD oxygen atom is a donor of the hydrogen bond, we can observe a similar tendency—the charge at OD is lower in the closed conformation than in the open one. This behaviour can be explained by the formation of the hydrogen bond, due to which the distance between OA and H shrinks, while the distance between OD and H grows. A significant redistribution of the charge density at the hydrogen bridge atom occurs and, as a consequence, the partial charge on both electronegative atoms lowers, while the hydrogen becomes more depleted in electron density. The models with the polarizable environment, whose impact was simulated in the study by using the PCM method, show that for both conformations the atomic charges present at the OD and OA atoms decrease, whereas atomic charges at the hydrogen atoms increase. The previous argument is also valid for the description of the system embedded in the PCM; one may also observe that usage of the PCM increases the polarizability of the atoms and leads to a less uniform distribution of the atomic charges and thus to an increase in the strength of the hydrogen bonds.

### 3.5. Natural Bond Orbitals and Wiberg Bond Indices (WBI) Analysis of Hydrogen Bonds of Monomers in the Gas Phase and in the PCM

**Figure 3:**
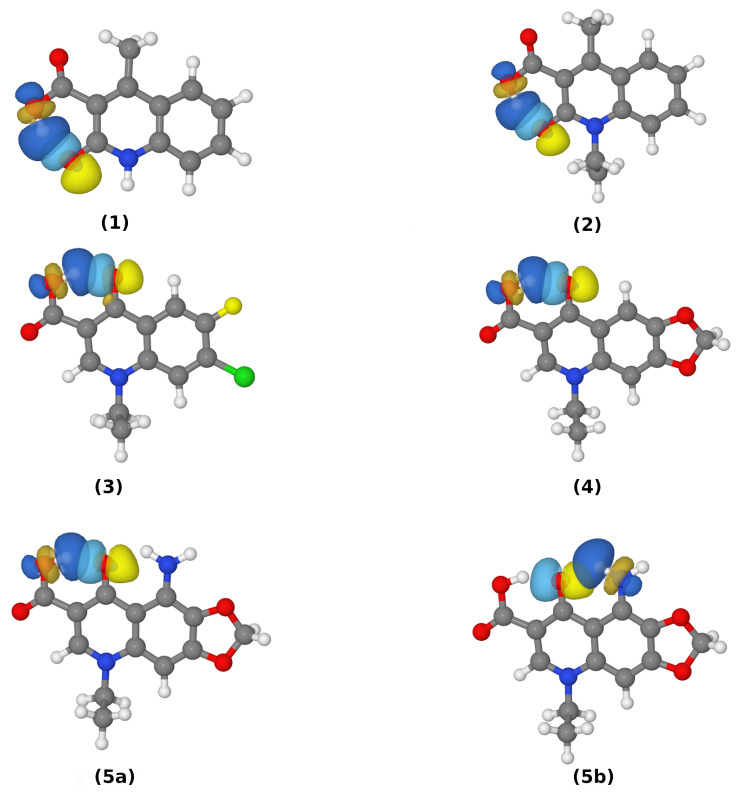
Natural bond orbitals of oxygen atoms LP(2) and the respective O–H/N–H $\sigma^{*}$ orbitals (N–H in the case of Compound **5** containing two intramolecular hydrogen bonds). The **5a** and **5b** labels indicate the presence of two intramolecular HBs in Compound **5**.

The NBO analysis provided more details into the hydrogen bond features for the monomers in different environments. The interaction energies of the lone pair (LP2) from the oxygen acceptor atom and the antibonding $\sigma^{*}$ orbital of O–H are provided in Table 4 and a graphical presentation of the overlapping orbitals is presented in Figure 3.

The overlapping of these orbitals, as well as the analysis of the data gathered in Table 4, show that intramolecular hydrogen bonds are present. Furthermore, the data shown in Table 4 provide evidence that the interaction energies between the antibonding $\sigma^{*}$ O–H orbital and the LP2 of the OA acceptor atom are significantly larger than for its LP1 counterpart with $\sigma^{*}$ O–H. In the gas phase, the interaction energies for the LP2-$\sigma^{*}$ O–H were larger by about five to seven times than the energies of the LP1-$\sigma^{*}$ O–H. Noteworthy, however expected, are the corresponding energy differences in the PCM. In this case the differences were even larger, what can be attributed to the effect of the polar environment exerted on the studied molecules, which results in a more anisotropically distributed charge within the interacting atoms. Furthermore, the data on the N–H•••OA bond (that competes with OD–H5•••OA in Compound **5**) were gathered in Table 4. The energy values comparison for LP2(O)-$\sigma^{*}$ O–H with LP2(O)-$\sigma^{*}$, both in the gas phase and PCM, demonstrates that the hydrogen from the amino group interacts with the lone pair on the oxygen acceptor atom in a significantly weaker way than the hydroxyl group’s hydrogen does. Besides the NBO, the Wiberg bond indices were calculated. Many reports suggest that WBI are more precise in hydrogen bond estimations than in different schemes of population analyses, mainly due to their dependency on the basis sets used in the calculations [62]. The WBI can be understood as a measure of electron density shared by two atoms; it is thus another approach for calculating the bond orders and estimating the covalency and strength of the various interactions (in fact, WBI are often of similar magnitudes to the bond orders expected from valence bond theory [89]). The universal observation that can be made on the basis of Table S4 is as follows: the electron density shared by every studied pair of atoms is larger for the systems embedded in the PCM environment than for those in the gas phase. This observation is coherent with the rest of the obtained data (see the respective subsections on AIM and NPA). Furthermore, as the data suggest, the substituent effects have a significant impact on the WBI values and, on the basis of the WBI analysis, it is supposed that both compounds with an ethyl group at the N1 atom form the strongest hydrogen bonds. Moreover, the para arrangement of the nitrogen and oxygen atoms in the ring and the carbonyl group, respectively, significantly facilitates the strongest HB formation in the PCM, whereas the ortho arrangement of these atoms leads to the highest Wiberg index in the gas phase. All of the inspected pairs of Hn•••OA, when the hydrogen atom donor was the oxygen, had indices of similar magnitude, whereas the lowest values in the gas and solvent phases corresponded to the atoms involved in the N–H•••O hydrogen bond formation.

### 3.6. Interaction Energy in Dimers of Quinolone Acid Derivatives Based on Symmetry-Adapted Perturbation Theory (SAPT) and Atoms in Molecules (AIM) Approaches

**Figure 4:**
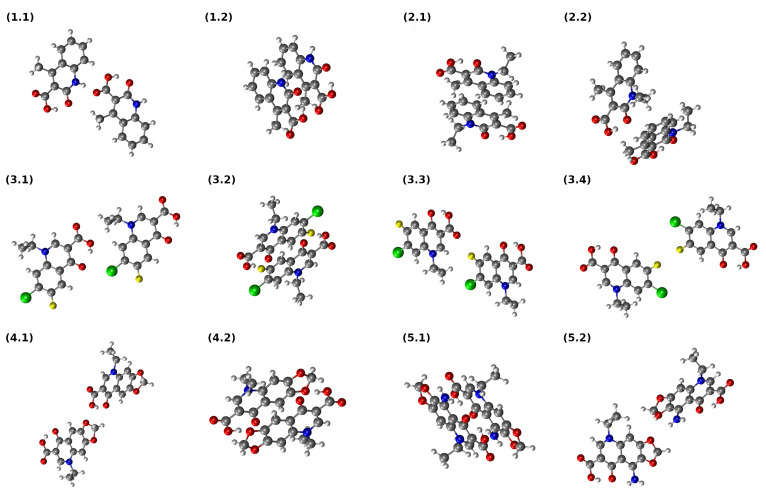
Dimers of the quinolone carboxylic acid derivatives extracted from the X-ray data and studied based on the SAPT. Atom colour coding: oxygen atoms, red; hydrogen atoms, white; carbon atoms, grey; chlorine atom, green; fluorine atoms, yellow; and nitrogen atoms, blue.

The self-assembly and self-organization processes of molecules are governed by the presence of non-covalent interactions, often differing widely in magnitude. Intermolecular HBs provide stabilization in the range of a few kcal mol^−1^ per single, highly directional contact. On the other hand, dispersion interactions are non-directional and much weaker, but ubiquitous. The molecules of the studied quinolone carboxylic acid derivatives are rich in non-covalent interaction centres—hydrogen bond donors and acceptors, as well as possible halogen bond sites (Compound **3** with its –F and –Cl functions), and, finally, extended aromatic systems interacting via dispersion. It is, therefore, not surprising that the examination of the crystal structures of **1**–**5** allowed us to recognize numerous types of homodimeric arrangements, gathered in Figure 4. The interaction energies for these dimers were evaluated at the SAPT2/aug-cc-pVDZ level of theory at both experimental (X-ray) and optimized (at the PBE-D3/aug-cc-pVDZ level, denoted further as DFT-D3) geometries. The results are gathered in Table 5 and the structures of the dimers based on the X-ray data are provided in Figure 4, while the DFT-D3 optimizations are summarized in Figure S2 of the Supplementary Material.

The dimers taken directly from the crystal structures exhibit a broad diversity of interaction types and corresponding interaction energy values. For Compound **1**, two selected structures, **1.1** and **1.2**, are interesting because, although they have very similar interaction SAPT2 energies—−10.093 and −11.527 kcal/mol, respectively—they differ greatly in their binding mechanisms. While **1.1** has a strong contribution of electrostatic and induction terms, **1.2** is a typical, dispersion-held stacked structure. This distinction is also present after the DFT-D3 optimization. On the other hand, two chosen dimers of **2** from the crystal structure are held rather by dispersion, with a low contribution of Coulombic forces. The DFT-D3 optimization leads to the collapse of these structures, **2.1** and **2.2**, into virtually identical stacked dimers. The broader structural diversity of Compound **3** stems from its asymmetric substitution with halogen atoms. The structure of **3.2** is that of a stacked dimer, and its stability is also aided by a favourable anti-parallel arrangement of dipole moments. The remaining structures, **3.1**, **3.3** and **3.4**, also benefit from dispersion, but they are much more weakly bound, and the electrostatic terms are less favourable. Thus, DFT-D3 optimization also leads in these cases to stacked, dispersion-held dimers.

The dimeric structures **3.1**, **3.3** and **3.4** deserve an extended description. In the experimental X-ray structure of **3** [18], the monomers are linked by interesting intermolecular NCIs. The **3.1** dimer contains short C–Cl•••OA contact of 3.104 Å to the carbonyl group. In **3.3** there is a C–Cl•••H–C contact of 3.010 Å, and **3.4** contains a C–F•••F–C contact of 2.768 Å. The two former contacts can be considered halogen bonds, while the last one is electrostatically unfavourable and the fluorine atoms usually do not exhibit such F•••F contacts. The value of the electrostatic term for **3.4**, −0.356 kcal/mol, is in principle stabilizing, but very low considering that the two monomers are arranged in the favourable anti–parallel geometry.

An extension of the quinolone skeleton by a third ring in **4** and **5** enhances the preference for dispersion-driven stacking. The non-stacked dimers **4.1** and **5.2** at their X-ray geometries are weakly bound (−4.052 and −1.657 kcal/mol, respectively) and cannot compete with the stacked dimers **4.2** and **5.1**, which are stabilized by more than 22 kcal/mol. The DFT-D3 optimization leads to the collapse of the non-stacked structures into the stacked structures.

As a summary of the SAPT interaction energy study, we note the dominant role of the dispersion in the studied dimers. We also note that the reasoning presented above is based on the more accurate SAPT2 perturbative expansion. The SAPT0 interaction energies, also given in Table 5, are qualitatively similar, with the exception of the **1.1** and **1.2** structures, which are differently ordered in their strength by SAPT0 and SAPT2.

An alternative approach to estimate the intermolecular interaction energies is provided by a topological analysis of the electron density field within the AIM theory. The simplest relation between the properties of the intermolecular BCP and the corresponding interaction energy is the Espinosa equation [85]. While its use in the intramolecular context is disputed [30], it is generally accepted in the intermolecular case [90,91,92,93,94]. In the current study, we applied this methodology to the selected non-equilibrated structures of dimers taken directly from the X-ray data. Diverse types of bonding are represented, ranging from conventional hydrogen bonds through weaker C–H•••O interactions and halogen bonds to dispersion-driven stacking. The topological AIM maps of the selected dimers are presented in Figure S3 of the Supplementary Material, while the relevant data (electron density, Laplacian, potential energy density and dissociation energy) are provided in Table S5. In general, the interaction energies dominated by hydrogen bonding are reproduced very consistently at the SAPT2 level and by the AIM-based Espinosa model. For example, the dimer **1.1** with a SAPT2 interaction energy of −10.093 kcal/mol is predicted to have a dissociation energy of 9.90 kcal/mol within the AIM-based scheme. The dimer **3.1** contains a halogen bond and three C–H•••O interactions; the Espinosa equation underestimates its stability by ca. two times as compared to the SAPT2 result. The dimer **3.3** is stabilized mostly by the halogen bonding, and its interaction energy is again described similarly by the SAPT2 and AIM. In the case of the dimer **3.4**, we noticed unusual C–F•••F–C contact, which seems to be overestimated by the Espinosa equation due to the electron-rich lone-pair regions of the fluorine atoms. The SAPT2 treats the dimer in a comprehensive way, taking into account the general distribution of charge density. The disagreement between the SAPT and AIM results is evident for the stacked dimer **5.1**, which simply points out the fact that the Espinosa equation was not designed to work with such weak, but multiple dispersion-driven, contacts. Finally, the dimer **5.2** is stabilized by both conventional N–H•••O and weak C–H•••H–C contacts, and the energy of the latter is overestimated within the AIM framework. In general, the obtained results indicate that the two different approaches, a comprehensive SAPT2 model and localized Espinosa equation, come to agree when the interactions are dominated by hydrogen/halogen bonds, whereas in cases in which dispersion plays a major role, the Espinosa equation cannot recover the total interaction energy.

### 3.7. Geometric and Spectroscopic Characteristics of the Intramolecular Hydrogen Bonds Based on Car–Parrinello Molecular Dynamics (CPMD)

**Figure 5:**
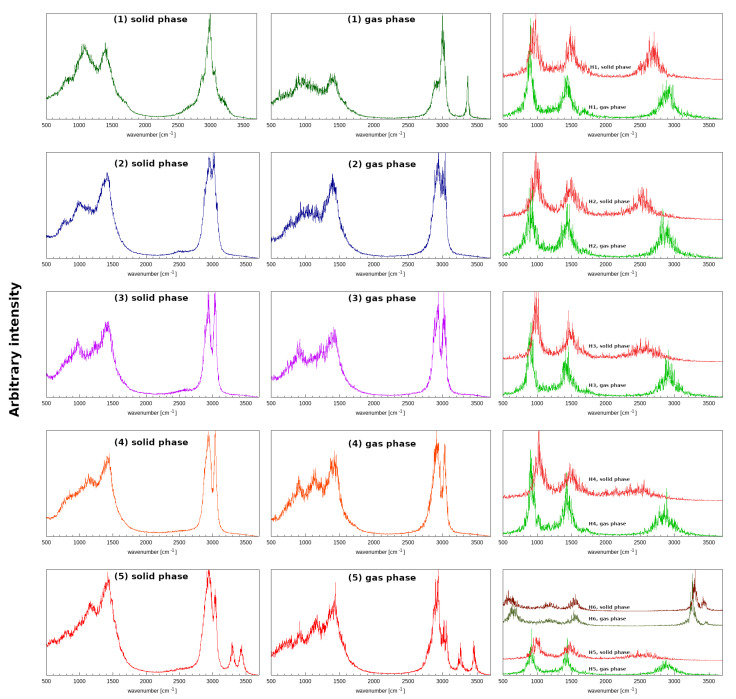
Atomic velocity power spectra of the studied quinolone carboxylic acid derivatives as results of the CPMD simulations. **Left panel**: the whole atom spectra in the solid state. **Middle panel**: the whole atom spectra in the gas phase. **Right panel**: the contribution of the bridged proton in the gas and solid phases.

In order to provide an insight into the systems behaviour as a function of time, the Car–Parrinello molecular dynamics method was employed, with special attention paid to the intramolecular hydrogen bonds present in the examined structures. The time evolution of the geometric parameters of the hydrogen bridges, thoroughly examined for the gas and crystalline phases, is presented in Figure S4. The gas-phase data (upper row of Figure S4) indicates that the distance between the proton acceptor and the proton itself varied significantly throughout the whole simulation. Generally, the hydrogen bond length varied between 1.5 and 2 Å, and the particularly large oscillations (even 2.5–1.5 Å) occurred for Compounds **1**, **2** and **4**. However, the obtained bond length OD–H1 and the interatomic distance OD•••OA suggest that proton-sharing events were not observed (the proton was located at the donor side). Different data were obtained for the models in the crystalline phase, where the Hn•••OA bond lengths were, on the whole, smaller than in the gas phase, and a higher mobility of the bridge protons was observed for every studied model. For Compound **4** the data also suggest that the proton sharing occurred with the frequency of ca. 1 sharing event every 10 ps of the simulation. Furthermore, one can notice that the proton resided at the acceptor side for a very brief period of time (ca. 1 ps) and subsequently returned to the proton–donor side. It can be observed that the length of our CPMD simulations (87 ps) was of the utmost importance, due to the fact that many proton-sharing events and the largest oscillations in the Hn•••OA bond lengths were seen in the final phases of our in silico experiment. In conclusion, the comparison between the data obtained for all of the molecules throughout the molecular dynamics in the gas phase and in the crystalline phase shows that the substituents that differ in the studied compounds and environmental effects such as other molecules in the vicinity, crystal field and the intermolecular interactions play an important role in proton dynamics characteristics. Another important part of the analysis included the calculations of the power spectra of the atomic velocity autocorrelation function, which enabled us to obtain the classical vibrational spectrum (Figure 5).

**Figure 6:**
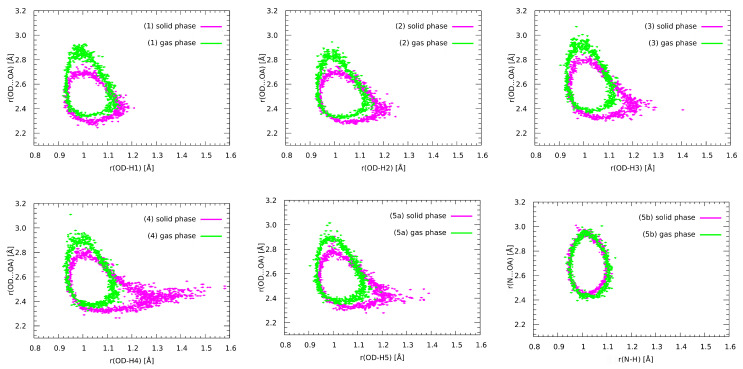
Relationships between the OD•••OA and OD–Hn distances for the examined compounds in the gas and solid phases. Probability density isocontours drawn at the 1 Å${}^{-2}$ value.

One of the biggest advantages of the employed methodology is its ability to easily estimate the individual contributions of specified atoms—in this case, particular attention was paid to the hydrogen atoms taking part in hydrogen bond formation. It is important to remember that the CPMD does not provide a full quantum description of the system, due to the fact that the nuclei are treated classically throughout the CPMD simulations—in consequence, phenomena such as tunnelling, overtones or Fermi resonances cannot be studied in this way. On the whole, the vibrational spectra of the gas and solid phases had two regions of increased intensity, namely: the region between 600 and 1800 cm^−1^ that was virtually identical in both phases between all of the studied structures, and the region between ca. 2100 to 3000 cm^−1^ in the solid phase and 2500 to 3100 cm^−1^ in the gas phase. The first region can be attributed to the oscillations of heavy atoms, whereas the second region of absorption is the signature region for bridge-protons motions. Experimental data on the vibrational features of the studied compounds are scarce. An IR and Raman study of fluoroquinolones [95] includes oxolinic acid **4**, but the spectra are discussed only up to 1800 cm^−1^. A study on rare-earth metal complexes of oxolinic acid [9] reports the IR spectrum of **4** in KBr pellets, and a broad absorption region in the 2200–3600 cm^−1^ range is registered. This agrees very well with the CPMD findings in the lower wavenumber region. Returning to the CPMD results, the presence of the crystal field allows for more frequent proton-sharing events (which was mentioned, when the geometrical features of hydrogen bridges were discussed) and thus results in a stronger anharmonicity of the potential energy surface, which makes the OH stretching modes more spread out and shifted to the lower wavenumbers. As can be observed in the third column of Figure 5, important differences between the studied compounds and thus the significance of the substituent effects on the proton dynamics can also be observed. For Compounds **1** and **2** the spread and shift of the OH stretches were less pronounced than for Compounds **3**–**5**. Furthermore, the structure of **4** shows the largest redshift (the redshift is usually attributed to the charge transfer processes from the proton acceptor to the $\sigma^{*}$ of the OH molecular orbital) and spread of bridge-proton bands, which accords very well with the hydrogen bond geometries and bridge-proton mobility observed in Figure S4. It can thus be regarded as evidence for stronger hydrogen bond formation in this case as well [96]. In the case of Compound **5**, the O–H band spreading and redshift in response to the presence of the crystal field was accompanied by a blueshift and more localized behaviour of the N–H band. One may interpret this behaviour as a sign of a weakening of the N–H•••OA bond and a strengthening of the OD–H•••OA bond that competes with the former. Furthermore, the probability distribution of the proton positions in the respective bridges, shown in Figure 6, provides a more intuitive way to understand the dynamics of the studied systems. The presence of proton-sharing events in the solid-phase simulations of **3**–**5**, especially in **4**, results in the characteristic “tail” extending towards the larger OD–Hn lengths and towards the HB midpoint. In this case, the histograms of Figure 6 are important indicators of the proton mobility.

### 3.8. Nuclear Quantum Effects Inclusion—An Application of Path Integral Molecular Dynamics (PIMD)

**Figure 7:**
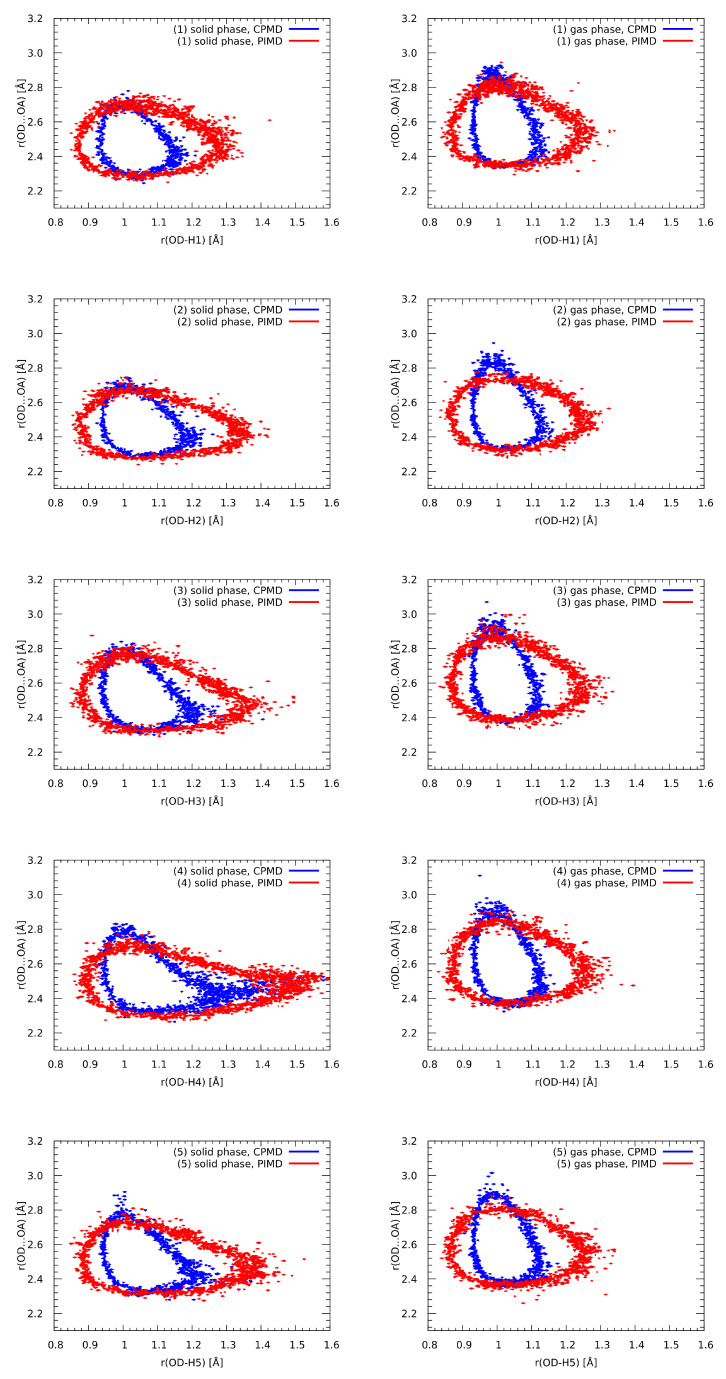
Impact of nuclear quantum effects on the OD•••OA and OD–Hn distances—comparison of the CPMD and PIMD simulations. Probability density isocontours drawn at the 1 Å${}^{-2}$ value.

The quantum description of a molecular system is usually carried out at the level of the electronic structure, while the nuclei are treated as classical particles. This separation within adiabatic and Born–Oppenheimer approximations is satisfactory in most cases, but lighter nuclei, especially protons, are liable to quantum effects. Hydrogen-bonded systems have been shown to benefit from the quantum description of protons as well as heavy-atom skeletons [97]. With this in mind, we performed path integral molecular dynamics (PIMD) simulations of **1**–**5** in the gas and crystalline phases. The PIMD does not correspond to the real-time quantum dynamics of the system—it yields quantum corrections to the statistical properties. Therefore, the PIMD results, presented in Figure 7 and compared against the CPMD runs, are shown in the form of a probability distribution of the proton in the OD-Hn•••OA bridge.

Inclusion of the quantum effects can result in qualitative or only quantitative changes of the proton behaviour. In the studied cases, the changes are of a quantitative nature. The proton position in the hydrogen bridge is shifted towards the midpoint between the donor and acceptor atoms, but there is no shift of equilibrium to the acceptor side—the proton-transfer form is not favoured. Quantum effects are most pronounced in the gas phase, because a polar crystal field already contributes to the shift of the proton position towards the bridge midpoint. The presence of the polar environment was essential for the possibility of short-lived proton-sharing events in the solid-phase simulations of **3**–**5**. Such events are even more probable when nuclear quantum effects are included, and even the weaker HBs of **1** and **2** could exhibit this phenomenon in the quantum regime. An important result was that the heavy-atom skeleton was affected by the quantum effects only to a small degree—the OD•••OA distance extends up to smaller (by 0.1 Å) values in PIMD than in the CPMD (for example, in the gas-phase simulation of **1** not up to 2.9 Å but only to 2.8 Å). The region of the lowest OD•••OA distances (2.35–2.4 Å) was not affected. The reason in this case might be that the acceptor OA atom was directly bound to the rigid ring skeleton, and the cost of lowering the HB length was prohibitive. Summarizing the results of the PIMD simulations, one has to note that the extent of proton delocalization was significantly enlarged for the weaker hydrogen bonds in the gas phase, while the solid-phase cases, especially Compounds **3**–**5**, were less affected. The proton participating in the hydrogen bond was able to sample more efficiently the potential energy surface, especially towards the acceptor region, and this resulted in a small, but noticeable, shortening of the HB length.

## 4. Conclusions

Five selected quinolone carboxylic acid derivatives with differently located carboxylic donor and carbonyl acceptor groups were studied in monomeric, dimeric and crystalline forms. The intramolecular OD–Hn•••OA hydrogen bonds formed between these groups can be divided into two classes of different strength: ca. 6.7 kcal/mol for **1** and **2**, and ca. 10 kcal/mol for **3**–**5**. As one can observe, the para arrangement of carbonyl group and the nitrogen heteroatom is a decisive factor in the formation of stronger hydrogen bonds. The polar solvent strengthens the HBs, which was shown from the points of view of both energetic and electronic structure (AIM, NBO and Wiberg bond index). The SAPT analysis indicated the decisive role of the non-covalent dispersive forces in the formation of the intermolecular dimers. The dynamical nature of the systems was studied through the CPMD approach. The proton-sharing events were recorded for the strongest HBs in the solid phase. The vibrational features of the O–H stretching mode showed that the crystal field and the vicinity of other molecules have a significant effect on the HB geometries and result in a shift of the $\nu_{OH}$ band location. The inclusion of quantum nuclear effects provided an accurate depiction of the bridged proton delocalization. However, the proton-transfer phenomena in the studied compounds are not favourable in the electronic ground state. The investigated compounds, well-recognized as medicines (oxolinic acid) or suitable for further derivatization studies in drug design, are also interesting from the physico-chemical point of view as examples of tunable intra- and intermolecular non-covalent interactions. 

## Figures and Tables

**Table 1:** CCDC code and unit cell data for the five studied quinolone carboxylic acid derivatives.

Number	CCDC Code	Unit Cell Data
**1**	PONNAI [10]	orthorhombic, a = 12.233 Å, b = 18.849 Å, c = 3.816 Å, Z = 4
**2**	MIRJIH [17]	monoclinic, a = 9.086 Å, b = 14.27 Å, c = 9.164 Å, β = 112.44${}^{\circ }$, Z = 4
**3**	EVINIG [18]	triclinic, a = 7.145 Å, b = 8.915 Å, c = 9.375 Å,
		α = 71.896${}^{\circ }$, β = 80.025${}^{\circ }$, γ = 85.109${}^{\circ }$, Z = 2
**4**	DAHWEO [11]	monoclinic, a = 7.182 Å, b = 10.575 Å, c = 14.758 Å, β = 94.26${}^{\circ }$, Z = 4
**5**	AMOXAC [12]	monoclinic, a = 7.485 Å, b = 10.449 Å, c = 15.087 Å, β = 99.69${}^{\circ }$, Z = 4

**Table 2:** Relative hydrogen bond energies as a difference of total energies of closed and open forms. Energy values given in kcal/mol.

System	H-Bond Energy	
	Gas Phase	PCM
**H1•••OA**	−6.738	−6.792
**H2•••OA**	−6.548	−6.623
**H3•••OA**	−9.300	−9.749
**H4•••OA**	−10.410	−10.994
**H5•••OA**	−9.880	−10.839

**Table 3:** NPA atomic charges for the atoms involved in the intramolecular hydrogen bond formation computed at the PBE-D3/aug-cc-pVTZ level of theory.

System	Atom	Closed		Open	
		Gas Phase	PCM	Gas Phase	PCM
**(1)**	**OD**	−0.634	−0.663	−0.630	−0.638
	**H1**	0.482	0.480	0.487	0.503
	**OA**	−0.616	−0.650	−0.570	−0.645
**(2)**	**OD**	−0.636	−0.664	−0.634	−0.641
	**H2**	0.483	0.480	0.486	0.502
	**OA**	−0.629	−0.652	−0.583	−0.646
**(3)**	**OD**	−0.636	−0.666	−0.617	−0.637
	**H3**	0.486	0.483	0.483	0.497
	**OA**	−0.598	−0.637	−0.532	−0.610
**(4)**	**OD**	−0.640	−0.672	−0.620	−0.640
	**H4**	0.485	0.479	0.480	0.495
	**OA**	−0.613	−0.658	−0.548	−0.630
**(5)**	**OD**	−0.640	−0.672	−0.664	−0.640
	**H5**	0.486	0.482	0.514	0.494
	**OA**	−0.664	−0.685	−0.611	−0.651

**Table 4:** Interaction energy (E2) between the lone pairs of the oxygen acceptor atom and the antibonding $\sigma^{*}$ (O–H) orbital. The **5a** and **5b** labels indicate the presence of two intramolecular HBs in Compound **5**. All energies given in kcal/mol.

System	LP1(O) $\to \sigma^{*}$ (O–H)	LP2(O) $\to \sigma^{*}$ (O–H)	LP1(O) $\to \sigma^{*}$ (O–H)	LP2(O) $\to \sigma^{*}$ (O–H)
	Gas Phase		PCM	
**(1)**	5.13	31.16	6.06	41.55
**(2)**	5.68	31.82	6.73	43.81
**(3)**	3.64	24.79	4.62	35.25
**(4)**	3.91	27.80	5.02	41.77
**(5a)**	5.12	25.67	5.93	38.70
**System**	LP1(O) $\to \sigma^{*}$ (N–H)	LP2(O) $\to \sigma^{*}$ (N–H)	LP1(O) $\to \sigma^{*}$ (N–H)	LP2(O) $\to \sigma^{*}$ (N–H)
	**Gas Phase**		**PCM**	
**(5b)**	4.88	4.58	4.40	3.05

**Table 5:** SAPT interaction energy partitioning obtained at the SAPT2/aug-cc-pVDZ and SAPT0/aug-cc-pVDZ levels of theory (kcal/mol) for dimeric structures (see Figure 4) taken from the X-ray data and optimized at the PBE-D3/aug-cc-pVDZ level of theory in the gas phase. The first digit in the structure code designates the studied compound and the second digit denotes the dimer type, as defined in Figure 4.

Dimer	Electrostatics	Exchange	Induction	Dispersion	SAPT0	SAPT2
**X-ray**						
**1.1**	−13.210	13.839	−5.403	−5.319	−13.214	−10.093
**1.2**	−2.750	14.970	−2.247	−21.499	−10.480	−11.527
**2.1**	−8.162	15.608	−2.094	−24.792	−20.580	−19.441
**2.2**	0.271	5.170	−1.133	−9.472	−5.184	−5.165
**3.1**	−6.450	5.900	−1.326	−5.147	−8.112	−7.023
**3.2**	−10.802	20.367	−3.908	−28.922	−23.880	−23.265
**3.3**	−1.243	3.386	−1.018	−4.433	−4.048	−3.308
**3.4**	−0.356	0.964	−0.0973	−1.218	−0.654	−0.707
**4.1**	−3.852	5.105	−1.029	−4.276	−4.782	−4.052
**4.2**	−10.309	14.439	−2.250	−24.401	−23.429	−22.522
**5.1**	−13.617	19.235	−3.448	−28.603	−27.730	−26.432
**5.2**	−1.084	4.779	−0.843	−4.510	−1.976	−1.657
**Optimized**						
**1.1**	−14.922	15.117	−6.299	−6.816	−17.099	−12.919
**1.2**	−6.548	14.830	−2.251	−20.270	−15.391	−14.239
**2.1**	−13.272	22.225	−4.115	−28.418	−26.131	−23.580
**2.2**	−12.823	23.481	−4.167	−28.621	−25.345	−22.129
**3.1**	−17.348	21.589	−4.866	−27.716	−30.881	−28.340
**3.2**	−12.679	22.836	−5.124	−31.014	−26.965	−25.981
**3.3**	−12.996	20.380	−4.500	−26.143	−24.953	−23.260
**3.4**	−10.035	20.814	−4.169	−27.937	−22.061	−21.327
**4.1**	−12.958	21.469	−3.736	−29.782	−26.509	−25.008
**4.2**	−21.296	29.549	−6.947	−31.403	−32.960	−30.096
**5.1**	−24.132	32.511	−7.157	−36.786	−38.720	−35.564
**5.2**	−22.811	31.007	−6.949	−33.766	−35.697	−32.518

## Data Availability

The data presented in the current study are available in the article and in the associated Supplementary Material.

## Supplementary Materials

The following are available online at https://www.mdpi.com/article/10.3390/molecules27072299/s1. Figure S1. Molecular structures of the studied quinolone carboxylic acid derivatives. The dotted line indicates the presence of an intramolecular hydrogen bond. Atom colour coding: oxygen atoms, red; hydrogen atoms, white; carbon atoms, grey; chlorine atom, green; fluorine atom, orange; and nitrogen atoms, blue. Table S1. Metric parameters of the intramolecular hydrogen bond. Comparison of experimental (X-ray) and computed data at the PBE-D3/aug-cc-pVTZ level of theory. Metric parameters are given in Å and (°). For the notation of hydrogen bridge atoms, see Figure S1. Table S2. AIM atomic charges of the O–H•••O intramolecular hydrogen bond computed in vacuo and with a solvent reaction field (PCM and water as a solvent) at the PBE-D3/aug-cc-pVTZ level of theory. Table S3. Bond critical points (BCPs) obtained for the intramolecular hydrogen bridges in the studied quinolone carboxylic acid derivatives. Electron density $\rho_{BCP}$ is given in $e\;\cdot \;a_{0}^{-3}$ atomic units, and its Laplacian $\nabla^{2}\rho_{BCP}$ in $e\;\cdot \;a_{0}^{-5}$ units. Table S4. Wiberg bond indices calculated based on natural atomic orbitals (NAO) at the PBE-D3/aug-cc-pVTZ level of theory. Figure S2. Dimers of the quinolone carboxylic acid derivatives obtained as a result of the geometry optimization at the PBE-D3/aug-cc-pVDZ level of theory studied based on the SAPT. Atom colour coding: oxygen atoms, red; hydrogen atoms, white; carbon atoms, grey; chlorine atom, green; fluorine atoms, yellow; and nitrogen atoms, blue. Figure S3. Topological maps of electron density based on AIM theory for the selected dimers of the quinolone carboxylic acid derivatives at the experimental X-ray geometry. Small green spheres denote bond critical points (BCPs); small red spheres denote ring critical points (RCPs). Atom colour coding: oxygen atoms, red; hydrogen atoms, white; carbon atoms, grey; chlorine atom, green; fluorine atom, lime green; and nitrogen atoms, blue. Table S5. Bond critical points (BCPs) obtained for the intermolecular contacts in the selected dimers of the studied quinolone carboxylic acid derivatives at the experimental (X-ray) geometry. Electron density $\rho_{BCP}$ is given in $e\;\cdot \;a_{0}^{-3}$ atomic units, and its Laplacian $\nabla^{2}\rho_{BCP}$ in $e\;\cdot \;a_{0}^{-5}$ units. The potential energy density V${}_{BCP}$ is given in hartree $e\;\cdot \;a_{0}^{-3}$ atomic units, while the corresponding dissociation energy D${}_{e,BCP}$ is given in kcal/mol according to the Espinosa equation. The atoms marked with an apostrophe are located in the second monomer of the dimeric system. Figure S4. Time evolution of atoms involved in intramolecular hydrogen bond formation. Top panel: CPMD results of the gas phase. Bottom panel: CPMD results of the solid state.

## Abbreviations

The following abbreviations are used in this manuscript:

DFT	Density functional theory
HB	Hydrogen bond
PCM	Polarizable continuum model
AIM	Atoms in molecules
NBO	Natural bond orbital
SAPT	Symmetry-adapted perturbation theory
CPMD	Car–Parrinello molecular dynamics
PIMD	Path integral molecular dynamics
NCI	Non-covalent interactions
CCDC	Cambridge Crystallographic Data Centre
PES	Potential energy surface
BCP	Bond critical point
RCP	Ring critical point
CCP	Cage critical point
RI	Resolution of identity
JKI	Coulomb and exchange integrals
BSSE	Basis set superposition error
NPA	Natural population analysis
NAO	Natural atomic orbital
WBI	Wiberg bond index
IR	Infrared spectroscopy

## References

[1] Mitscher L.A. (2005). Bacterial Topoisomerase Inhibitors: Quinolone and Pyridone Antibacterial Agents. Chem. Rev..

[2] Dayam R., Al-Mawsawi L.Q., Zawahir Z., Witvrouw M., Debyser Z., Neamati N. (2008). Quinolone 3-Carboxylic Acid Pharmacophore: Design of Second Generation HIV-1 Integrase Inhibitors. J. Med. Chem..

[3] Horta P., Kuş N., Henriques M.S.C., Paixão J.A., Coelho L., Nogueira F., O’Neill P.M., Fausto R., Cristiano M.L.S. (2015). Quinolone–Hydroxyquinoline Tautomerism in Quinolone 3-Esters. Preserving the 4-Oxoquinoline Structure To Retain Antimalarial Activity. J. Org. Chem..

[4] Hong W.D., Leung S.C., Amporndanai K., Davies J., Priestley R.S., Nixon G.L., Berry N.G., Hasnain S.S., Antonyuk S., Ward S.A. (2018). Potent Antimalarial 2-Pyrazolyl Quinolone bc1 (Qi) Inhibitors with Improved Drug-like Properties. ACS Med. Chem. Lett..

[5] Tan L., Zhang Z., Gao D., Chan S., Luo J., Tu Z.C., Zhang Z.M., Ding K., Ren X., Lu X. (2019). Quinolone antibiotic derivatives as new selective Axl kinase inhibitors. Eur. J. Med. Chem..

[6] Gong M., Yang Y., Huang Y., Gan T., Wu Y., Gao H., Li Q., Nie J., Huang W., Wang Y. (2021). Novel quinolone derivatives targeting human dihydroorotate dehydrogenase suppress Ebola virus infection in vitro. Antiviral Res..

[7] Andersson M.I. (2003). Development of the quinolones. J. Antimicrob. Chemother..

[8] Calvo A., Giménez M., Alou L., Gómez-Lus M., Aguilar L., Prieto J. (2002). Ex vivo serum activity (killing rates) after gemifloxacin 320 mg versus trovafloxacin 200 mg single doses against ciprofloxacin-susceptible and -resistant Streptococcus pneumoniae. Int. J. Antimicrob. Agents.

[9] Maciuca A.M., Munteanu A.C., Mihaila M., Badea M., Olar R., Nitulescu G.M., Munteanu C.V.A., Bostan M., Uivarosi V. (2020). Rare-Earth Metal Complexes of the Antibacterial Drug Oxolinic Acid: Synthesis, Characterization, DNA/Protein Binding and Cytotoxicity Studies. Molecules.

[10] Ukrainets I.V., Bereznyakova N.L., Parshikov V.A., Kravchenko V.N. (2008). 4-Hydroxy-2-quinolones 140. Synthesis and diuretic activity of arylalkylamides of 4-methyl-2-oxo-1, 2-dihydro-quinoline-3-carboxylic acid. Chem. Heterocycl. Compd..

[11] Cygler M., Huber C.P. (1985). Structure of oxolinic acid, a potent antibacterial agent. 1-Ethyl-1, 4-dihydro-6, 7-methylenedioxy-4-oxo-3-quinolinecarboxylic acid, C_13_H_11_NO_5_. Acta Cryst. C.

[12] Czugler M., Argay G., Frank J., Mészáros Z., Kutschabsky L., Reck G. (1976). 1-Ethyl-1, 4-dihydro-4-oxo-5-amino-6, 7-methylenedioxy-3-quinolinecarboxylic acid (aminooxolinic acid). Acta Cryst. B.

[13] Gleckman R., Alvarez S., Joubert D.W., Matthews S.J. (1979). Drug therapy reviews: Oxolinic acid. Am. J. Hosp. Pharm..

[14] Tuma J., Connors W.H., Stitelman D.H., Richert C. (2002). On the Effect of Covalently Appended Quinolones on Termini of DNA Duplexes. J. Am. Chem. Soc..

[15] Irgi E.P., Geromichalos G.D., Balala S., Kljun J., Kalogiannis S., Papadopoulos A., Turel I., Psomas G. (2015). Cobalt(ii) complexes with the quinolone antimicrobial drug oxolinic acid: Structure and biological perspectives. RSC Adv..

[16] Mjos K.D., Cawthray J.F., Polishchuk E., Abrams M.J., Orvig C. (2016). Gallium(iii) and iron(iii) complexes of quinolone antimicrobials. Dalton Trans..

[17] Ukrainets I.V., Bereznyakova N.L., Parshikov V.A., Shishkina S.V. (2007). 4-hydroxy-2-quinolones. 114. Synthesis and structure of 6-R-5-hydroxy-2,4-dioxo-2,3,4,6-tetrahydrobenzo-[c][2,7]naphthyridine-1-carbonitriles. Chem. Heterocycl. Compd..

[18] Song J., Xue F.H., Lou K.X. (2004). 7-Chloro-1-ethyl-6-fluoro-1,4-dihydro-4-oxoquinoline-3-carboxylic acid. Acta Cryst. E.

[19] Cleland W.W., Kreevoy M.M. (1994). Low-Barrier Hydrogen Bonds and Enzymic Catalysis. Science.

[20] Wu Z.R., Ebrahimian S., Zawrotny M.E., Thornburg L.D., Perez-Alvarado G.C., Brothers P., Pollack R.M., Summers M.F. (1997). Solution Structure of 3-Oxo-Δ^5^-Steroid Isomerase. Science.

[21] Remer L.C., Jensen J.H. (2000). Toward a General Theory of Hydrogen Bonding: The Short, Strong Hydrogen Bond [HOH···OH]-. J. Phys. Chem. A.

[22] Lane J.R. (2012). CCSDTQ Optimized Geometry of Water Dimer. J. Chem. Theory Comput..

[23] Alcock N. (1972). Secondary Bonding to Nonmetallic Elements. Advances in Inorganic Chemistry and Radiochemistry.

[24] Grabowski S.J. (2011). What Is the Covalency of Hydrogen Bonding?. Chem. Rev..

[25] Jeffrey G., Saenger W. (1991). Hydrogen Bonding in Biological Structures.

[26] Jeffrey G. (1997). An Introduction to Hydrogen Bonding.

[27] Mautner M.M.N. (2005). The Ionic Hydrogen Bond. Chem. Rev..

[28] Gilli P., Bertolasi V., Ferretti V., Gilli G. (1994). Evidence for resonance-assisted hydrogen bonding. 4. Covalent nature of the strong homonuclear hydrogen bond. Study of the O-H–O system by crystal structure correlation methods. J. Am. Chem. Soc..

[29] Gilli P., Bertolasi V., Pretto L., Ferretti V., Gilli G. (2004). Covalent versus Electrostatic Nature of the Strong Hydrogen Bond: Discrimination among Single, Double, and Asymmetric Single-Well Hydrogen Bonds by Variable-Temperature X-ray Crystallographic Methods in *β*-Diketone Enol RAHB Systems. J. Am. Chem. Soc..

[30] Jabłoński M. (2020). A Critical Overview of Current Theoretical Methods of Estimating the Energy of Intramolecular Interactions. Molecules.

[31] Grabowski S. (2001). An estimation of strength of intramolecular hydrogen bonds—ab initio and AIM studies. J. Mol. Struct..

[32] Lane J.R., Schrøder S.D., Saunders G.C., Kjaergaard H.G. (2016). Intramolecular Hydrogen Bonding in Substituted Aminoalcohols. J. Phys. Chem. A.

[33] Pauling L. (1960). The Nature of the Chemical Bond and the Structure of Molecules and Crystals; an Introduction to Modern Structural Chemistry.

[34] Cavallo G., Metrangolo P., Milani R., Pilati T., Priimagi A., Resnati G., Terraneo G. (2016). The Halogen Bond. Chem. Rev..

[35] Kolář M.H., Hobza P. (2016). Computer Modeling of Halogen Bonds and Other *σ*-Hole Interactions. Chem. Rev..

[36] Costa P.J. (2017). The halogen bond: Nature and applications. Phys. Sci. Rev..

[37] Riley K.E., Tran K.A. (2017). Strength, character, and directionality of halogen bonds involving cationic halogen bond donors. Faraday Discuss..

[38] Engelage E., Reinhard D., Huber S.M. (2020). Is There a Single Ideal Parameter for Halogen-Bonding-Based Lewis Acidity?. Chem. Eur. J..

[39] Portela S., Fernández I. (2021). Nature of the Hydrogen Bond Enhanced Halogen Bond. Molecules.

[40] Dang Q.M., Simpson J.H., Parish C.A., Leopold M.C. (2021). Evaluating Halogen-Bond Strength as a Function of Molecular Structure Using Nuclear Magnetic Resonance Spectroscopy and Computational Analysis. J. Phys. Chem. A.

[41] Auffinger P., Hays F.A., Westhof E., Ho P.S. (2004). Halogen bonds in biological molecules. Proc. Natl Acad. Sci. USA.

[42] Brinck T., Murray J.S., Politzer P. (1992). Surface electrostatic potentials of halogenated methanes as indicators of directional intermolecular interactions. Int. J. Quant. Chem..

[43] Hohenberg P., Kohn W. (1964). Inhomogeneous Electron Gas. Phys. Rev..

[44] Kohn W., Sham L.J. (1965). Self-Consistent Equations Including Exchange and Correlation Effects. Phys. Rev..

[45] Bader R. (1994). Atoms in Molecules: A Quantum Theory.

[46] Foster J.P., Weinhold F. (1980). Natural hybrid orbitals. J. Am. Chem. Soc..

[47] Weinhold F., Landis C.R. (2001). Natural Bond Orbitals and extensions of localized bonding concepts. Chem. Educ. Res. Pract..

[48] Weinhold F., Landis C.R. (2012). Discovering Chemistry with Natural Bond Orbitals.

[49] Jeziorski B., Moszynski R., Szalewicz K. (1994). Perturbation Theory Approach to Intermolecular Potential Energy Surfaces of van der Waals Complexes. Chem. Rev..

[50] Car R., Parrinello M. (1985). Unified Approach for Molecular Dynamics and Density-Functional Theory. Phys. Rev. Lett..

[51] Marx D., Parrinello M. (1996). The Effect of Quantum and Thermal Fluctuations on the Structure of the Floppy Molecule C_2_H_3_^+^. Science.

[52] Tuckerman M.E., Marx D., Klein M.L., Parrinello M. (1996). Efficient and general algorithms for path integral Car–Parrinello molecular dynamics. J. Chem. Phys..

[53] (2021). CCDC Structural Database. https://www.ccdc.cam.ac.uk/.

[54] Mercury—Crystal Structure Visualisation. http://www.ccdc.cam.ac.uk/Solutions/CSDSystem/Pages/Mercury.aspx.

[55] Miertuš S., Scrocco E., Tomasi J. (1981). Electrostatic interaction of a solute with a continuum. A direct utilizaion of AB initio molecular potentials for the prevision of solvent effects. Chem. Phys..

[56] Cossi M., Barone V., Cammi R., Tomasi J. (1996). Ab initio study of solvated molecules: A new implementation of the polarizable continuum model. Chem. Phys. Lett..

[57] Perdew J.P., Burke K., Ernzerhof M. (1996). Generalized Gradient Approximation Made Simple. Phys. Rev. Lett..

[58] Grimme S. (2006). Semiempirical GGA-type density functional constructed with a long-range dispersion correction. J. Comput. Chem..

[59] Dunning T.H. (1989). Gaussian basis sets for use in correlated molecular calculations. I. The atoms boron through neon and hydrogen. J. Chem. Phys..

[60] Frisch M.J., Trucks G.W., Schlegel H.B., Scuseria G.E., Robb M.A., Cheeseman J.R., Scalmani G., Barone V., Petersson G.A., Nakatsuji H. (2016). Gaussian~16 Revision A.03.

[61] Keith T.A. (2019). AIMAll (Version 19.10.12), TK Gristmill Software.

[62] Wiberg K. (1968). Application of the Pople-Santry-Segal CNDO method to the cyclopropylcarbinyl and cyclobutyl cation and to bicyclobutane. Tetrahedron.

[63] Glendening E.D., Reed A.E., Carpenter J.E., Weinhold F. NBO Version 3.1. https://www.scienceopen.com/document?vid=6652d352-0292-499f-88d6-2221dae56281.

[64] Glendening E.D., Landis C.R., Weinhold F. (2013). NBO 6.0: Natural bond orbital analysis program. J. Comput. Chem..

[65] Frisch M.J., Trucks G.W., Schlegel H.B., Scuseria G.E., Robb M.A., Cheeseman J.R., Scalmani G., Barone V., Mennucci B., Petersson G.A. (2010). Gaussian 09, Revision C.01.

[66] (2016). Jmol: An Open-Source Java Viewer for Chemical Structures in 3D, Version 14.6.4. https://sourceforge.net/p/jmol/.

[67] Patek M. (2015). Jmol NBO Visualization Helper, Version 2.1. https://www.marcelpatek.com/blgdownload/JmolNboVHelper2.1.zip.

[68] Hohenstein E.G., Sherrill C.D. (2010). Density fitting of intramonomer correlation effects in symmetry-adapted perturbation theory. J. Chem. Phys..

[69] Kendall R.A., Dunning T.H., Harrison R.J. (1992). Electron affinities of the first-row atoms revisited. Systematic basis sets and wave functions. J. Chem. Phys..

[70] Boys S., Bernardi F. (1970). The calculation of small molecular interactions by the differences of separate total energies. Some procedures with reduced errors. Mol. Phys..

[71] Smith D.G.A., Burns L.A., Simmonett A.C., Parrish R.M., Schieber M.C., Galvelis R., Kraus P., Kruse H., Di Remigio R., Alenaizan A. (2020). PSI4 1.4: Open-source software for high-throughput quantum chemistry. J. Chem. Phys..

[72] Schlegel H.B. (1984). Estimating the hessian for gradient-type geometry optimizations. Theor. Chem. Acc..

[73] Hockney R.W. (1970). Potential Calculation and Some Applications. Methods Comput. Phys..

[74] Hoover W.G. (1985). Canonical dynamics: Equilibrium phase-space distributions. Phys. Rev. A.

[75] Troullier N., Martins J.L. (1991). Efficient pseudopotentials for plane-wave calculations. Phys. Rev. B.

[76] Humphrey W., Dalke A., Schulten K. (1996). VMD—Visual Molecular Dynamics. J. Mol. Graph..

[77] Williams T., Kelley C., Bersch C., Sebald D., Campbell J., Cunningham R., Denholm D., Elber G., Fearick R., Grammes C. (2019). Gnuplot 5.2.8: An Interactive Plotting Program. http://www.gnuplot.info.

[78] CPMD Version 4.3-4610, Copyright IBM Corp (1990–2004) Copyright MPI für Festkoerperforschung Stuttgart (1997–2001). http://www.cpmd.org/.

[79] Backus J.W., Stern H., Ziller I., Hughes R.A., Nutt R., Beeber R.J., Best S., Goldberg R., Haibt L.M., Herrick H.L. (1957). The FORTRAN automatic coding system. IRE-AIEE-ACM ’57 (Western): Papers Presented at the 26–28 February 1957, Western Joint Computer Conference: Techniques for Reliability.

[80] Filarowski A., Koll A., Głowiak T. (2002). Proton transfer equilibrium in the intramolecular hydrogen bridge in sterically hindered Schiff bases. J. Mol. Struct..

[81] Ukrainets I.V., Sidorenko L.V., Gorokhova O.V., Shishkina S.V. (2006). 4-hydroxy-2-quinolones. 96. Synthesis and properties of 4-methyl-2-oxo-1,2-dihydroquinoline-3-carboxylic acid. Chem. Heterocycl. Compd..

[82] Ukrainets I.V., Gorokhova O.V., Sidorenko L.V., Bereznyakova N.L. (2007). 4-Hydroxy-2-quinolones. 111. Simple synthesis of 1-substituted 4-methyl-2-oxo-1,2-dihydroquinoline-3-carboxylic acids. Chem. Heterocycl. Compd..

[83] Dixit S.K., Yadav N., Kumar S., Good L., Awasthi S.K. (2014). Synthesis and antibacterial activity of novel fluoroquinolone analogs. Med. Chem. Res..

[84] Koch U., Popelier P.L.A. (1995). Characterization of C-H-O Hydrogen Bonds on the Basis of the Charge Density. J. Phys. Chem..

[85] Espinosa E., Molins E., Lecomte C. (1998). Hydrogen bond strengths revealed by topological analyses of experimentally observed electron densities. Chem. Phys. Lett..

[86] Grabowski S.J., Sokalski W.A., Dyguda E., Leszczyński J. (2006). Quantitative Classification of Covalent and Noncovalent H-Bonds. J. Phys. Chem. B.

[87] Reed A.E., Weinstock R.B., Weinhold F. (1985). Natural population analysis. J. Chem. Phys..

[88] Martin F., Zipse H. (2004). Charge distribution in the water molecule—A comparison of methods. J. Comput. Chem..

[89] Cao Q. (2013). Dinitroamino benzene derivatives: A class new potential high energy density compounds. J. Mol. Model..

[90] Mondal P.K., Yadav H.R., Choudhury A.R., Chopra D. (2017). Quantitative characterization of new supramolecular synthons involving fluorine atoms in the crystal structures of di- and tetrafluorinated benzamides. Acta Crystallogr. B Struct. Sci. Cryst. Eng. Mater..

[91] Al-Mutairi A.A., Katari B.K.P., Narasimhan Y., Blacque O., Al-Wahaibi L.H., Al-Alshaikh M.A., El-Emam A.A., Percino M.J., Thamotharan S. (2020). Interplay of weak intermolecular interactions in two Schiff’s bases with organic fluorine derived from 5-nitrothiophene-2-carboxaldehyde: Crystal structures, DFT calculation and in vitro evaluation of bioactivities. J. Mol. Struct..

[92] El-Emam A.A., Kumar E.S., Janani K., Al-Wahaibi L.H., Blacque O., El-Awady M.I., Al-Shaalan N.H., Percino M.J., Thamotharan S. (2020). Quantitative assessment of the nature of noncovalent interactions in N-substituted-5-(adamantan-1-yl)-1,3,4-thiadiazole-2-amines: Insights from crystallographic and QTAIM analysis. RSC Adv..

[93] Tariq S., Khalid M., Raza A.R., Rubab S.L., de Alcântara Morais S.F., Khan M.U., Tahir M.N., Braga A.A.C. (2020). Experimental and computational investigations of new indole derivatives: A combined spectroscopic, SC-XRD, DFT/TD-DFT and QTAIM analysis. J. Mol. Struct..

[94] Khalid M., Ali A., Asim S., Tahir M.N., Khan M.U., Vieira L.C.C., de la Torre A.F., Usman M. (2021). Persistent prevalence of supramolecular architectures of novel ultrasonically synthesized hydrazones due to hydrogen bonding [X–H⋯O; X=N]: Experimental and density functional theory analyses. J. Phys. Chem. Solids.

[95] Neugebauer U., Szeghalmi A., Schmitt M., Kiefer W., Popp J., Holzgrabe U. (2005). Vibrational spectroscopic characterization of fluoroquinolones. Spectrochim. Acta A Mol. Biomol. Spectrosc..

[96] Hansen P.E., Spanget-Larsen J. (2017). NMR and IR Investigations of Strong Intramolecular Hydrogen Bonds. Molecules.

[97] Tuckerman M.E., Marx D. (2001). Heavy-Atom Skeleton Quantization and Proton Tunneling in “Intermediate-Barrier” Hydrogen Bonds. Phys. Rev. Lett..

