# Supplementary material for: Revealing Intra- and Intermolecular Interactions Determining Physico-Chemical Features of Selected Quinolone Carboxylic Acid Derivatives
Source: Molecules. 2022 Apr 1;27(7):2299. doi: 10.3390/molecules27072299 (PMC9000753; doi:10.3390/molecules27072299)
Supplement: Supplementary file 1 [file molecules-27-02299-s001.zip › molecules-1647898-supplementary.pdf]

SUPPLEMENTARY MATERIAL

Revealing Intra- and Intermolecular Interactions Determining Physico-chemical Features of Selected Quinolone Carboxylic Acid Derivatives

Kamil Wojtkowiak[1], Aneta Jezierska[1], Jarosław J. Panek[1]

[1] University of Wrocław, Faculty of Chemistry, ul. F. Joliot Curie 14, 50-383 Wrocław, Poland

Correspondence should be addressed to: aneta.jezierska@chem.uni.wroc.pl; jaroslaw.panek@chem.uni.wroc.pl

Table of contents:

I. **Figure S1.** Molecular structures of the studied quinolone carboxylic acid derivatives. Dotted line indicates the presence of an intramolecular hydrogen bond. Atom color coding: oxygen atoms – red, hydrogen atoms – white, carbon atoms – grey, chlorine atom – green, fluorine atom – orange and nitrogen atoms – blue.

II. **Table S1.** Metric parameters of the intramolecular hydrogen bond. Comparison of experimental (X-ray) and computed data at the PBE-D3/aug-cc-pVTZ level of theory. Metric parameters are given in [Å] and [°]. For hydrogen bridge atoms notation see Figure S1.

III. **Table S2.** AIM atomic charges of the O-H…O intramolecular hydrogen bond computed in vacuo and with solvent reaction field (PCM and water as a solvent) at the PBE-D3/aug-cc-pVTZ level of theory.

IV. **Table S3.** Bond Critical Points (BCPs) obtained for the intramolecular hydrogen bridges in the studied quinolone carboxylic acid derivatives. Electron density ρ_{BCP} is given in $e*a_0^{-3}$ atomic units, and its Laplacian $\nabla^2\rho_{BCP}$ in $e*a_0^{-5}$ units.

V. **Table S4.** Wiberg bond indices calculated based on Natural Atomic Orbitals (NAO) at the PBE-D3/aug-cc-pVTZ level of theory.

VI. **Figure S2.** Dimers of the quinolone carboxylic acid derivatives obtained as a result of the geometry optimization at the PBE-D3/aug-cc-pVDZ level of theory studied based on the SAPT. Atom color coding: oxygen atoms -red, hydrogen atoms - white, carbon atoms - grey, chlorine atom - green, fluorine atoms - yellow and nitrogen atoms - blue.

VII. **Figure S3.** Topological maps of electron density based on AIM theory for the selected dimers of the quinolone carboxylic acid derivatives at the experimental X-ray geometry. Small green spheres denote Bond Critical Points (BCPs), small red spheres denote Ring Critical Points (RCPs). Atom color coding: oxygen atoms – red, hydrogen atoms – white, carbon atoms – grey, chlorine atom – green, fluorine atom – lime green and nitrogen atoms – blue.

VIII. **Table S5.** Bond Critical Points (BCPs) obtained for the intermolecular contacts in the selected dimers of the studied quinolone carboxylic acid derivatives at the experimental (X-ray) geometry. Electron density ρ_{BCP} is given in $e{*}a_0^{-3}$ atomic units, and its Laplacian $\nabla^2\rho_{BCP}$ in $e{*}a_0^{-5}$ units. The potential energy density V_{BCP} is given in hartree$*a_0^{-3}$ atomic units, while the corresponding dissociation energy $D_{e,BCP}$ is given in kcal/mol according to the Espinosa equation. The atoms marked with apostrophe are located in the monomer 2 of the dimeric system.

IX. **Figure S4.** Time evolution of atoms involved in the intramolecular hydrogen bond formation. Top panel: CPMD results of the gas phase. Bottom panel: CPMD results of the solid state.

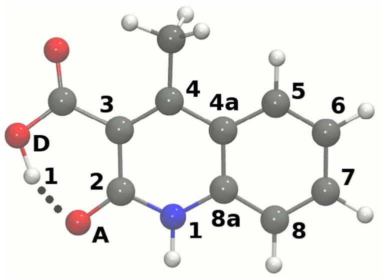

(1)

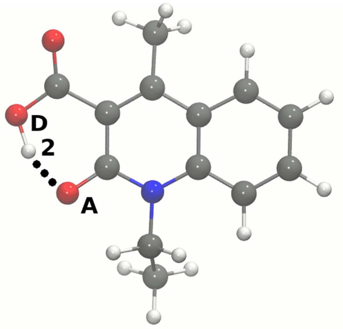

(2)

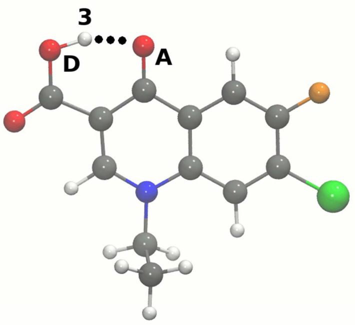

(3)

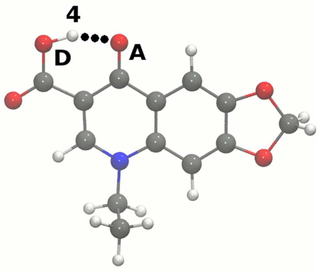

(4)

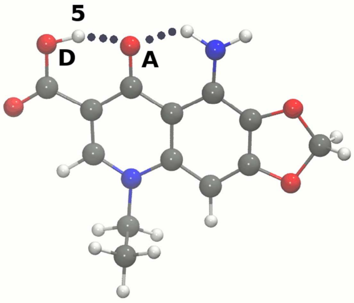

(5)

Figure S1. Molecular structures of the studied quinolone carboxylic acid derivatives. Dotted line indicates the presence of an intramolecular hydrogen bond. Atom color coding: oxygen atoms – red, hydrogen atoms – white, carbon atoms – grey, chlorine atom – green, fluorine atom – orange and nitrogen atoms – blue.

Table S1. Metric parameters of the intramolecular hydrogen bond. Comparison of experimental (X-ray) and computed data at the PBE-D3/aug-cc-pVTZ level of theory. Metric parameters are given in [Å] and [°]. For hydrogen bridge atoms notation see Figure S1.

**Metric parameters**	**Experimental X-ray data**	**PBE-D3/aug-cc-pVTZ (*in vacuo*)**	**PBE-D3/aug-cc-pVTZ (PCM)***
**Compound 1 Ref. [10]**			
**OD··OA**	**2.465**	**2.5196**	**2.4767**
**OD-H1**	**0.895**	**1.0172**	**1.0355**
**H1··OA**	**1.638**	**1.5616**	**1.4910**
**<ODH1OA**	**152.14**	**154.83**	**156.81**
**Compound 2 Ref. [17]**			
**OD··OA**	**2.451**	**2.5079**	**2.4622**
**OD-H2**	**0.940**	**1.0177**	**1.0387**
**H1··OA**	**1.548**	**1.5506**	**1.4734**
**<ODH2OA**	**159.8**	**154.5**	**156.8**
**Compound 3 Ref. [18]**			
**OD··OA**	**2.549**	**2.5868**	**2.5253**
**OD-H3**	**0.820**	**1.0158**	**1.0346**
**H1··OA**	**1.785**	**1.6306**	**1.5405**
**<ODH3OA**	**154.5**	**154.9**	**157.0**
**Compound 4 Ref. [11]**			
**OD··OA**	**2.545**	**2.5703**	**2.5025**
**OD-H4**	**0.964**	**1.0205**	**1.0453**
**H1··OA**	**1.648**	**1.6056**	**1.5020**
**<ODH4OA**	**153.0**	**155.7**	**158.1**
**Compound 5 Ref. [12]**			
**OD··OA**	**2.518**	**2.5627**	**2.4991**
**OD-H5**	**0.941**	**1.0172**	**1.0402**
**H1··OA**	**1.626**	**1.6027**	**1.5056**
**<ODH5OA**	**156.6**	**155.4**	**157.6**

***water was used as a solvent**

Table S1 (Continuation). Metric parameters of the intramolecular hydrogen bond. Comparison of experimental (X-ray) and computed data at the PBE-D3/aug-cc-pVTZ level of theory. Metric parameters are given in [Å] and [°]. For hydrogen bridge atoms notation see Figure S1.

**Metric parameters**	**Experimental X-ray data**	**PBE-D3/aug-cc-pVTZ (*in vacuo*)**	**PBE-D3/aug-cc-pVTZ (PCM)***
**Compound 5 Ref. [12]**			
**N··OA**	**2.664**	**2.6307**	**2.6608**
**N-H**	**0.865**	**1.0223**	**1.0193**
**H··OA**	**1.978**	**1.8343**	**1.8819**
**<NHOA**	**135.5**	**132.0**	**130.7**

***water was used as a solvent**

Table S2. AIM atomic charges of the O-H…O intramolecular hydrogen bond computed *in vacuo* and with solvent reaction field (PCM and water as a solvent) at the PBE-D3/aug-cc-pVTZ level of theory.

Atomic charge [e]	Gas phase	PCM
**Compound 1**		
**qOD**	**-1.1136**	**-1.1369**
**qH1**	**0.6254**	**0.6259**
**qOA**	**-1.1177**	**-1.1390**
**Compound 2**		
**qOD**	**-1.1159**	**-1.1391**
**qH2**	**0.6259**	**0.6261**
**qOA**	**-1.1179**	**-1.1331**
**Compound 3**		
**qOD**	**-1.1072**	**-1.1317**
**qH3**	**0.6191**	**0.6205**
**qOA**	**-1.0960**	**-1.1224**
**Compound 4**		
**qOD**	**-1.1110**	**-1.1366**
**qH4**	**0.6192**	**0.6186**
**qOA**	**-1.1045**	**-1.1335**

Table S2 (Continuation). AIM atomic charges computed *in vacuo* and with solvent reaction field (PCM and water as a solvent) at the PBE-D3/aug-cc-pVTZ level of theory.

**Atomic charge [e]**	**Gas phase**	**PCM**
**Compound 5**		
**qOD**	**-1.1126**	**-1.1381**
**qH5**	**0.6195**	**0.6207**
**qOA**	**-1.1173**	**-1.1318**
**qN**	**-1.1314**	**-1.1132**
**qH**	**0.4832**	**0.4658**
**qOA**	**-1.1173**	**-1.1318**

Table S3. Bond Critical Points (BCPs) obtained for the intramolecular hydrogen bridges in the studied quinolone carboxylic acid derivatives. Electron density ρ_{BCP} is given in $e*a_0^{-3}$ atomic units, and its Laplacian $\nabla^2\rho_{BCP}$ in $e*a_0^{-5}$ units.

BCP	Gas phase		PCM	
	ρ_{BCP}	$\nabla^2\rho_{BCP}$	ρ_{BCP}	$\nabla^2\rho_{BCP}$
**Compound 1**				
**OD-H1**	**0.3122**	**-2.1138**	**0.2949**	**-1.8894**
**H1···OA**	**0.0712**	**0.1045**	**0.0853**	**0.0909**
**Compound 2**				
**OD-H2**	**0.3117**	**-2.1050**	**0.2920**	**-1.8481**
**H2···OA**	**0.0731**	**0.1048**	**0.0891**	**0.0878**
**Compound 3**				
**OD-H3**	**0.3143**	**-2.1431**	**0.2965**	**-1.9174**
**H3···OA**	**0.0608**	**0.0975**	**0.0762**	**0.0879**
**Compound 4**				
**OD-H4**	**0.3098**	**-2.0868**	**0.2871**	**-1.7849**
**H4···OA**	**0.0648**	**0.0956**	**0.0841**	**0.0794**
**Compound 5**				
**OD-H5**	**0.3131**	**-2.1233**	**0.2915**	**-1.8441**
**H5···OA**	**0.0648**	**0.0988**	**0.0828**	**0.0846**
**N-H**	**0.3318**	**-1.9023**	**0.3361**	**-1.9002**
**H···OA**	**0.0372**	**0.1207**	**0.0333**	**0.1158**

Table S4. Wiberg bond indices calculated based on Natural Atomic Orbitals (NAO) at the PBE-D3/aug-cc-pVTZ level of theory.

Atom pair	Gas phase	PCM
**H1···OA**	**0.1283**	**0.1565**
**H2···OA**	**0.1296**	**0.1616**
**H3···OA**	**0.1143**	**0.1456**
**H4···OA**	**0.1237**	**0.1637**
**H5···OA**	**0.1172**	**0.1537**
**H···OA**	**0.0399**	**0.0328**

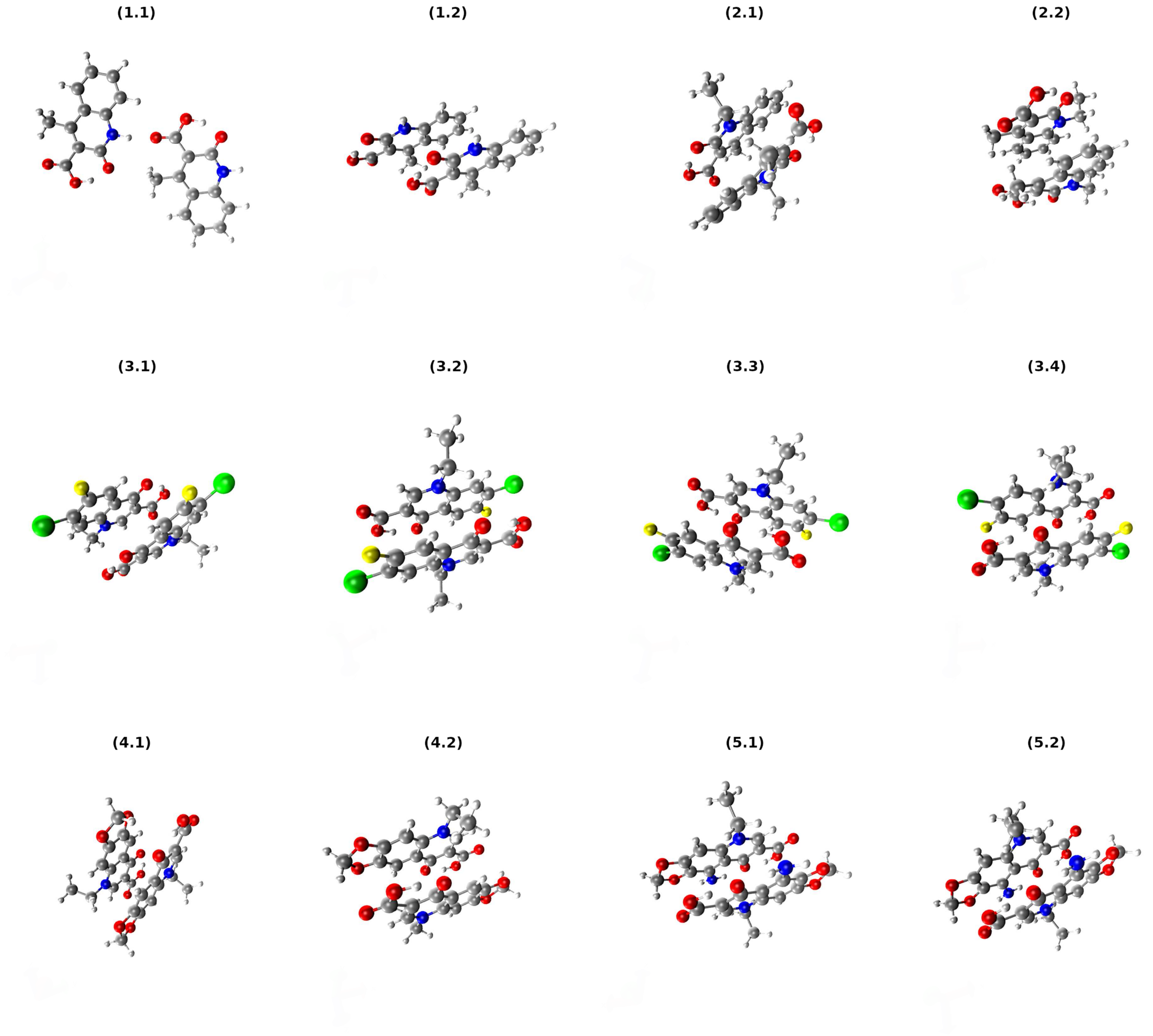

Figure S2. Dimers of the quinolone carboxylic acid derivatives obtained as a result of the geometry optimization at the PBE-D3/aug-cc-pVDZ level of theory studied based on the SAPT. Atom color coding: oxygen atoms -red, hydrogen atoms - white, carbon atoms - grey, chlorine atom - green, fluorine atoms - yellow and nitrogen atoms – blue.

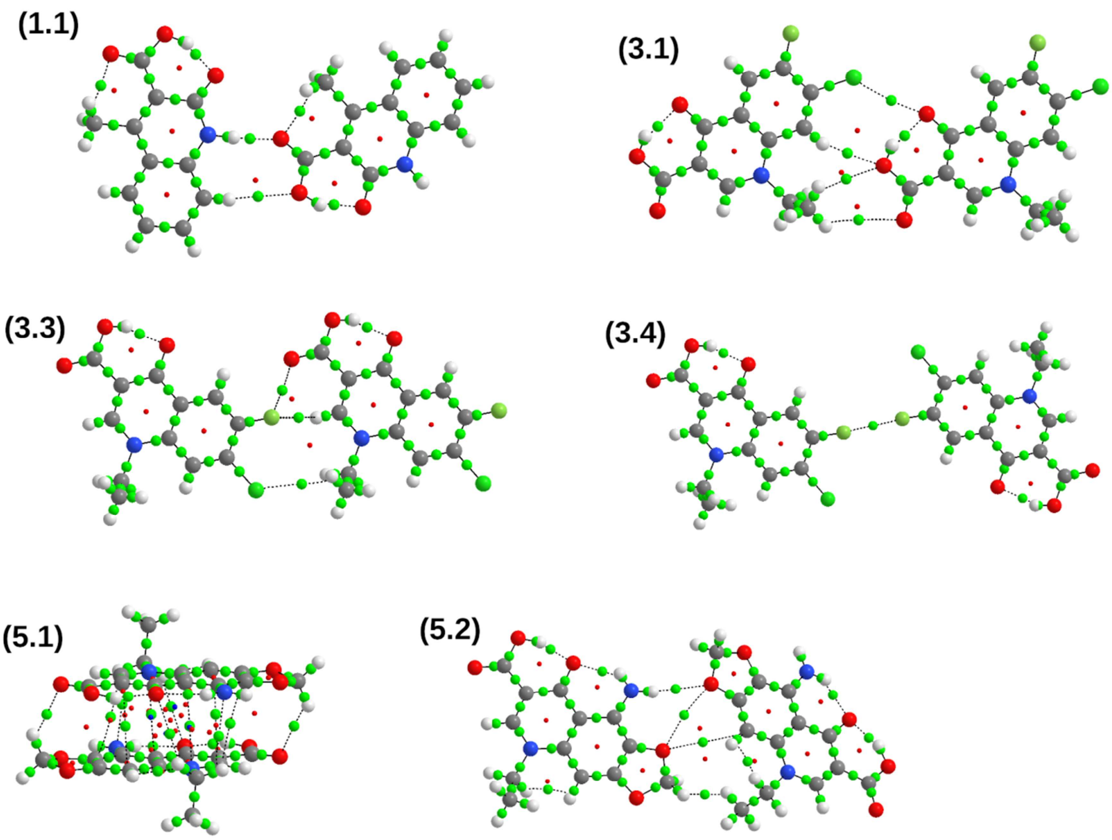

Figure S3. Topological maps of electron density based on AIM theory for the selected dimers of the quinolone carboxylic acid derivatives at the experimental X-ray geometry. Small green spheres denote Bond Critical Points (BCPs), small red spheres denote Ring Critical Points (RCPs). Atom color coding: oxygen atoms– red, hydrogen atoms – white, carbon atoms – grey, chlorine atom – green, fluorine atom – lime green and nitrogen atoms – blue.

Table S5. Bond Critical Points (BCPs) obtained for the intermolecular contacts in the selected dimers of the studied quinolone carboxylic acid derivatives at the experimental (X-ray) geometry. Electron density ρ_{BCP} is given in $e*a_0^{-3}$ atomic units, and its Laplacian $\nabla^2\rho_{BCP}$ in $e*a_0^{-5}$ units. The potential energy density V_{BCP} is given in $hartree*a_0^{-3}$ atomic units, while the corresponding dissociation energy $D_{e,BCP}$ is given in kcal/mol according to the Espinosa equation [85]. The atoms marked with apostrophe are located in the monomer 2 of the dimeric system.

Interaction:	ρ_{BCP}	$\nabla^2\rho_{BCP}$	V_{BCP}	$D_{e,BCP}$
**Dimer 1.1**				
**N-H··O'**	**0.0330**	**0.1061**	**-0.02964**	**9.30**
**C8-H··OD'**	**0.0039**	**0.0139**	**-0.00190**	**0.60**
			**sum:**	**9.90**
**Dimer 3.1**				
**C7-Cl··OA'**	**0.0085**	**0.0352**	**-0.00485**	**1.52**
**C8-H··OD'**	**0.0079**	**0.0314**	**-0.00441**	**1.38**
**C-H(ethyl)··OD'**	**0.0032**	**0.0123**	**-0.00154**	**0.48**
**C-H(ethyl)··O'**	**0.0020**	**0.0067**	**-0.00083**	**0.26**
			**sum:**	**3.64**
**Dimer 3.3**				
**C6-F··O'**	**0.0040**	**0.0144**	**-0.00234**	**0.73**
**C6-F··H'-C2'**	**0.0057**	**0.0239**	**-0.00348**	**1.09**
**C7-Cl··H'-C'(ethyl)**	**0.0058**	**0.0210**	**-0.00278**	**0.87**
			**sum:**	**2.69**
**Dimer 3.4**				
**C6-F··F'-C6'**	**0.0063**	**0.0348**	**-0.00478**	**1.50**
**Dimer 5.1**				
**multiple (12) contacts between the stacked molecules**	**0.0039 ÷ 0.0060**	**0.0126 ÷ 0.0214**	**-0.00157 ÷ -0.00311**	**sum: 7.83**
**Dimer 5.2**				
**N-H··O(ring)'**	**0.0060**	**0.0246**	**-0.00342**	**1.07**
**O(ring3)··O(ring3)'**	**0.0020**	**0.0076**	**-0.00100**	**0.31**
**O(ring3)··C8'**	**0.0017**	**0.0061**	**-0.00069**	**0.22**
**C(ring3)-H··H'-C'(ethyl)**	**0.0129**	**0.0375**	**-0.00690**	**2.16**
			**sum:**	**3.76**

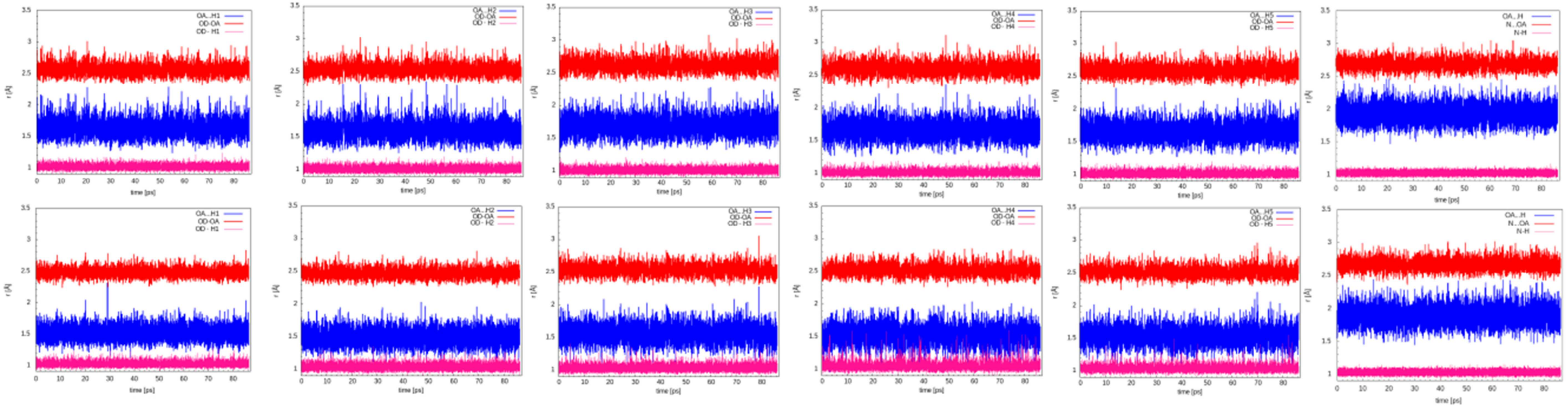

Figure S4. Time evolution of atoms involved in the intramolecular hydrogen bond formation. Top panel: CPMD results of the gas phase. Bottom panel: CPMD results of the solid state.
